# Investigating Europa’s Habitability with the Europa Clipper

**DOI:** 10.1007/s11214-023-01025-2

**Published:** 2023-11-29

**Authors:** Steven D. Vance, Kathleen L. Craft, Everett Shock, Britney E. Schmidt, Jonathan Lunine, Kevin P. Hand, William B. McKinnon, Elizabeth M. Spiers, Chase Chivers, Justin D. Lawrence, Natalie Wolfenbarger, Erin J. Leonard, Kirtland J. Robinson, Marshall J. Styczinski, Divya M. Persaud, Gregor Steinbrügge, Mikhail Y. Zolotov, Lynnae C. Quick, Jennifer E. C. Scully, Tracy M. Becker, Samuel M. Howell, Roger N. Clark, Andrew J. Dombard, Christopher R. Glein, Olivier Mousis, Mark A. Sephton, Julie Castillo-Rogez, Francis Nimmo, Alfred S. McEwen, Murthy S. Gudipati, Insoo Jun, Xianzhe Jia, Frank Postberg, Krista M. Soderlund, Catherine M. Elder

**Affiliations:** 1 Jet Propulsion Laboratory, California Institute of Technology, Pasadena, CA USA; 2 Applied Physics Laboratory, Johns Hopkins University, Laurel, MD USA; 3 School of Earth & Space Exploration and School of Molecular Sciences, Arizona State University, Tempe, AZ USA; 4 Department of Astronomy and Department of Earth & Atmospheric Sciences, Cornell University, Ithaca, NY USA; 5 Department of Earth and Planetary Sciences and McDonnell Center for the Space Sciences, Washington University in St. Louis, Saint Louis, MO USA; 6 School of Earth and Atmospheric Sciences, Georgia Institute of Technology, Atlanta, GA USA; 7 Applied Ocean Physics and Engineering, Woods Hole Oceanographic Institution, Woods Hole, MA USA; 8 Honeybee Robotics, Altadena, CA USA; 9 Institute for Geophysics, John A. and Katherine G. Jackson School of Geosciences, University of Texas at Austin, Austin, TX USA; 10 Beyond Center, Arizona State University, Tempe, AZ USA; 11 NASA Goddard Space Flight Center, Greenbelt, MD USA; 12 Southwest Research Institute, San Antonio, TX USA; 13 Planetary Science Institute, Tucson, AZ USA; 14 Dept. of Earth and Environmental Sciences, University of Illinois Chicago, Chicago, USA; 15 Aix Marseille Université, CNRS, LAM (Laboratoire d’Astrophysique de Marseille), Marseille, France; 16 Impacts and Astromaterials Research Centre, Department of Earth Science and Engineering, Imperial College London, London, United Kingdom; 17 Department of Earth and Planetary Sciences, University of California, Santa Cruz, CA USA; 18 Lunar and Planetary Laboratory, University of Arizona, Tucson, AZ USA; 19 Department of Climate and Space Sciences and Engineering, University of Michigan, Ann Arbor, MI USA; 20 Institut für Geologische Wissenschaften, Freie Universität Berlin, Berlin, Germany

**Keywords:** Europa, Ocean worlds, Ice, Habitability, Jupiter

## Abstract

The habitability of Europa is a property within a system, which is driven by a multitude of physical and chemical processes and is defined by many interdependent parameters, so that its full characterization requires collaborative investigation. To explore Europa as an integrated system to yield a complete picture of its habitability, the Europa Clipper mission has three primary science objectives: (1) characterize the ice shell and ocean including their heterogeneity, properties, and the nature of surface–ice–ocean exchange; (2) characterize Europa’s composition including any non-ice materials on the surface and in the atmosphere, and any carbon-containing compounds; and (3) characterize Europa’s geology including surface features and localities of high science interest. The mission will also address several cross-cutting science topics including the search for any current or recent activity in the form of thermal anomalies and plumes, performing geodetic and radiation measurements, and assessing high-resolution, co-located observations at select sites to provide reconnaissance for a potential future landed mission. Synthesizing the mission’s science measurements, as well as incorporating remote observations by Earth-based observatories, the James Webb Space Telescope, and other space-based resources, to constrain Europa’s habitability, is a complex task and is guided by the mission’s Habitability Assessment Board (HAB).

## Habitability of Europa

**Fig. 1:**
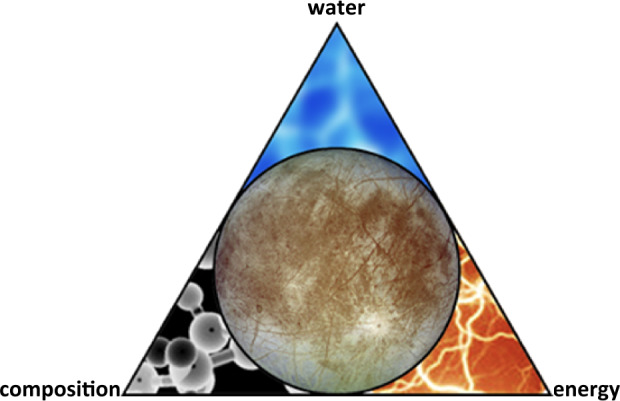
Jupiter’s moon Europa is one the most promising candidates for hosting life today among ocean worlds in the solar system. In its investigation of Europa’s habitability, the Europa Clipper mission seeks to understand the provenance of water, essential chemical elements and compounds, and energy, and how they might combine to make this moon’s environments suitable to support life. Modified from a pre-Europa Clipper mission study report (Europa Study Team 2012)

At its most basic, the Europa Clipper mission is conceived as an investigation of Europa’s water, essential chemical elements and compounds, and energy sources, all directed to better understand Europa’s potential habitability (Fig. 1). The mission will confirm (or conceivably, refute) the existence of Europa’s subsurface ocean, and it will further characterize the moon through multiple investigations in order to constrain its habitability. The goal of the Europa Clipper mission is not to detect life itself, but to assess Europa’s ability to support life as we know it (further addressed below) by harboring essential chemical compounds and sources of energy in addition to liquid water (e.g., Greenberg et al. 2000; Chyba and Phillips 2001; Cable et al. 2020). In this paper, we describe the context of Europa’s habitability as currently conceived, and how the Europa Clipper science team plans to achieve the synthesis of measurements needed to understand whether the moon is or was suitable to host life.

Compelling evidence for a present-day subsurface water ocean beneath Europa’s icy surface was first obtained by the Galileo mission through measurements of an induced magnetic field that was best fit by the existence of an electrically conductive layer, which was interpreted to be a salty, liquid water ocean in the near-surface interior (Kivelson et al. 1997, 1999, 2000; Khurana et al. 1998, 2009; Roberts et al. 2023). The existence of an ocean seemed plausible because substantial heat is generated inside Europa through tidal dissipation occurring during the moon’s eccentric orbit about Jupiter (e.g., Squyres et al. 1983; Ojakangas and Stevenson 1989; Soderlund et al. 2014). The orbital eccentricity causes internal friction and dissipation in its icy shell, and critically, within the moon’s rocky interior, where water–rock interactions may provide reductants and other essential chemicals required for habitability (e.g., Hsu et al. 2015; Běhounková et al. 2021). At Europa’s orbital distance from Jupiter, strong oxidants (O_2_, H_2_O_2_, etc.) are produced on the surface from the intense bombardment by energetic particles accelerated in the Jovian magnetosphere. If these oxidants are transported into the interior and mix with potentially hydrogen-rich reductants from the seafloor (Vance et al. 2016), the resulting chemical reactions may provide abundant and long-lasting energy for metabolism as we know it on the Earth (Hand et al. 2009).

This paper addresses the high-level question of “what is habitability?” and describes how the Europa Clipper mission will assess Europa’s habitability in particular. Section 2 comprises a major portion of the paper and discusses how Europa’s formation and evolution determine the energy and chemical elements available for habitability (and habitation), and details the measurements that will be made and what will potentially be learned from them. Specific potentially habitable environments on Europa (ice shell, ocean, and seafloor) are then discussed in Sect. 3. The paper concludes with a brief look-ahead to landed studies (Sect. 4) and summary remarks (Sect. 5).

### What Is Habitability?

The definition of habitability that pertains to the Europa Clipper’s assessment is this: A habitable environment contains the chemical ingredients and physical conditions clement for life, even life considered extreme on Earth. A habitable environment is not necessarily inhabited, i.e., survivable conditions do not imply the presence of life is present; however, based on our current understanding of life on Earth may sustain life in this environment. To grow and survive, known life requires certain chemical elements and compounds and physical conditions (e.g., Smith et al. 2021). Relevant parameters include a hospitable temperature, pressure, pH, salinity, and the presence of a solvent such as water (e.g., Schulze-Makuch and Irwin 2018; Hendrix et al. 2019; Styczinski et al. 2022 and references within). The combination of these properties, in the context of a long-lived physical setting, determines the types of organisms that might thrive.

Extremophiles are organisms that live in environments limited in one or more of the requirements for life (Pikuta et al. 2007), and these organisms define the bounds of known habitability, providing a guide for life’s possible limits. These limits constrain whether Europa can support life at all. This assessment is one that the Europa Clipper science team intends to perform. Subsequently, if Europa is deemed habitable, identifying the types and quantity of possible organisms that Europa might support is critical for planning future life detection missions. Any potential biosignatures detected by such a future mission (or conceivably, by Europa Clipper itself; Salter et al. 2022a,b) would need to be understood in terms of the physical and chemical conditions that produced them, including whether those conditions could produce detectable biosignatures (e.g., Lorenz 2016). Constraining the energetic fluxes between Europa’s reservoirs—both thermal and chemical—is required to understand the context of potential biosignatures. For example, excesses of a particular compound such as oxygen, in concert with another compound it reacts with such as methane, provide a sign of life on Earth known as a disequilibrium biosignature (Krissansen-Totton et al. 2016). Of course, for any potential biosignature, potential abiotic sources and their likelihood of producing or contributing to the signal should be rigorously evaluated as the default null hypothesis.

**Fig. 2:**
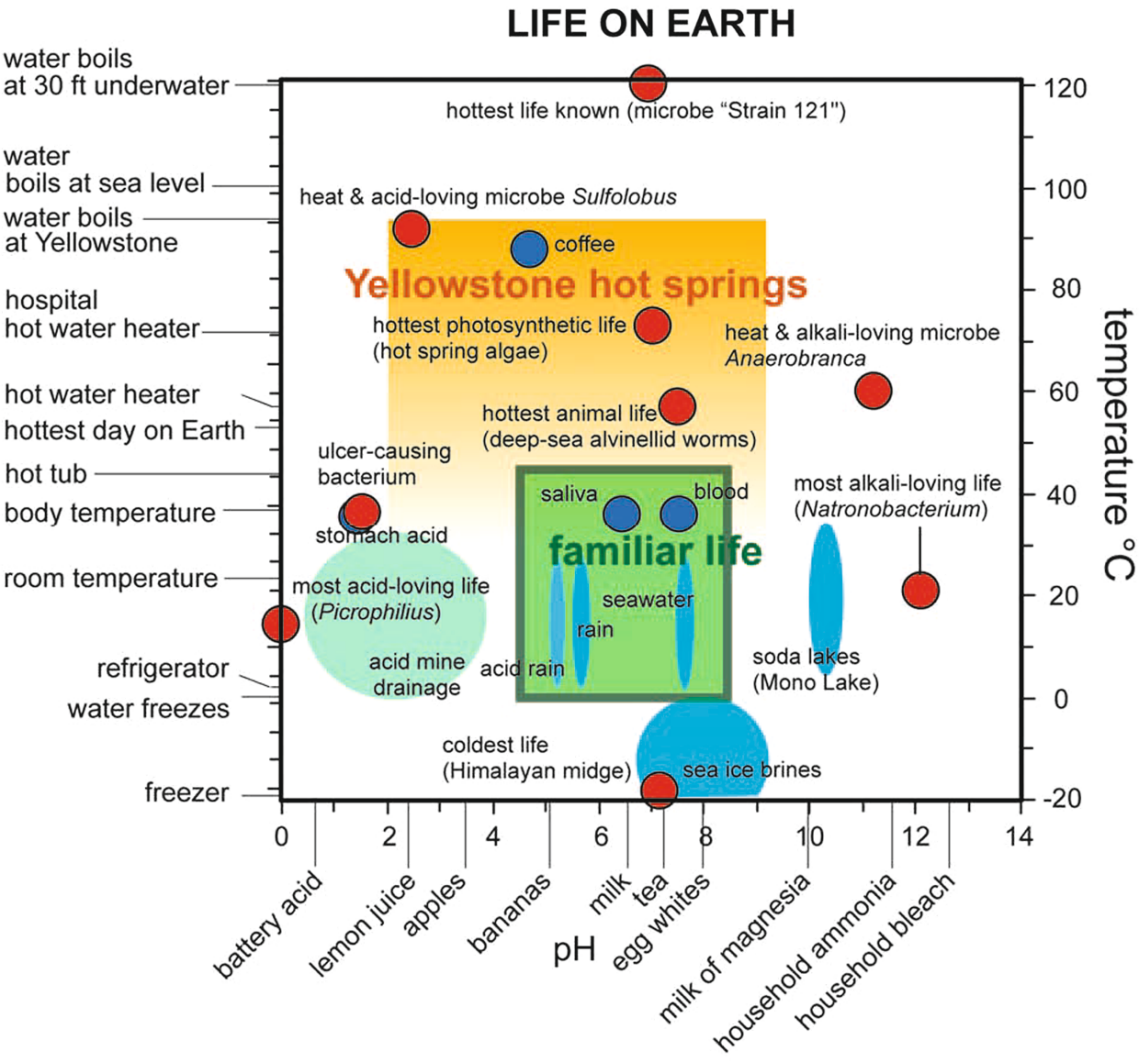
Examples of extremophiles and common environments on Earth for varying solution pH and temperature. The investigation of Europa’s habitability extends beyond the binary of whether or not life can survive there. On Earth, life can be found almost everywhere water is stable as a liquid. How much biomass is present—and thus how easily that life might be detected—depends on the available chemical energy, which varies mainly with conditions of temperature and pH, as shown here. In addition to confirming the presence of liquid water, Europa Clipper may be able to characterize the details of Europa’s habitability by identifying the specific environmental conditions that are present and how long they might have existed. Reproduced from Shock and Holland (2007)

The investigation of habitability can be thought of as revealing environmental conditions in the context of possible biological activity, as compared to signatures of abiotic cycles of chemical elements. In this view, a systems-level interpretation of Europa Clipper observations, measurements, and models could quantify the potential biomass at Europa (e.g., Affholder et al. 2021). For example, on Earth, a pH around 7, temperature in the range of 0 to 100 °C, and the easy availability of nutrients are hallmarks of a setting capable of supporting a large amount of biomass (Fig. 2), thereby likely providing sufficient material for study with scientific instruments. Though life can persist in a broader range of pH, and at lower temperatures, the amounts of biomass that can be supported at these more extreme conditions are typically much smaller on Earth and presumably would also be so on Europa. The kinetics of biomass degradation in such environments are also not well known, adding to uncertainties inherent in studying potential life in these different environments from any we know on Earth (Hoehler 2022). These considerations underscore the importance of quantifying and contextualizing habitability as well as providing critical context for any direct search for life.

### How Europa Clipper Will Assess Habitability

The overarching goal of NASA’s Europa Clipper mission is “to explore Europa to investigate its habitability” (Pappalardo et al. 2024 this collection). Because habitability is an emergent property of a complex system, as described above, with many interdependent physical and chemical parameters and processes, no single measurement or investigation can characterize Europa’s habitability. Instead, results from investigating Europa as a system must be integrated into a more complete picture. The Europa Clipper mission therefore has three primary science objectives to investigate Europa’s habitability: (1) characterize the ice shell and any subsurface water, including their heterogeneity, ocean properties, and the nature of surface–ice–ocean exchange; (2) identify the composition of any non-ice materials on the surface and in the atmosphere, including any carbon-containing compounds; and (3) characterize geological surface features and high-science-interest localities. The mission will also address several cross-cutting science topics by searching for any current or recent activity in the form of thermal anomalies and/or plumes, performing geodetical and radiation measurements, and assessing high-resolution, co-located observations at select sites to provide reconnaissance for a potential future landed mission. Each investigation within the Europa Clipper mission contributes to one or more of the three primary science objectives, and together they will achieve the mission’s goal to assess Europa’s habitability. In this respect, the Europa Clipper investigations and associated data are highly complementary, and the synthesis of Europa Clipper science extends beyond the individual measurement requirements for the mission.

To accomplish these objectives and cross-cutting assessments, Europa Clipper will use a suite of in situ and remote sensing investigations:

- Europa Clipper Magnetometer (ECM)
- Europa Imaging System (EIS) Narrow Angle Camera (NAC) and Wide Angle Camera (WAC)
- Europa THermal EMission Imaging System (E-THEMIS)
- Europa UltraViolet Spectrograph (Europa-UVS)
- Gravity and Radio Science (G/RS)
- MAss Spectrometer for Planetary EXploration (MASPEX)
- Mapping Imaging Spectrometer for Europa (MISE)
- Plasma Instrument for Magnetic Sounding (PIMS)
- Radar for Europa Assessment and Sounding: Ocean to Near-surface (REASON)
- SUrface Dust Analyzer (SUDA)

A radiation monitor is part of the engineering instrumentation and its multiple sensors will measure the spacecraft’s exposure to highly energetic particles. These data will aid in characterizing the radiation environment of Europa. Further, to optimize science data collection, the science instruments and engineering sensors have been carefully placed on the spacecraft to allow simultaneous data collection while pointing the remote-sensing instruments at the same surface locations and sampling the in situ environment above these regions. Additionally, the mission operations plan ensures science data return based on science priority and feed-forward instrument data needs, and the measurement plans, acquired data, and science results are discussed and shared across the full science team.

Planned measurements by the science investigations will be combined to achieve the Level 1 science requirements (L1s) and enable assessment of Europa’s habitability. Table 1 shows how each investigation’s contributions, by science theme, tie to achieving the L1s. Measurements build upon one another to inform about habitability. For example, observations of the surface materials provide information about the composition and conditions of the ocean and its interaction with the rocky mantle vs. exogenic contributions. These findings can then serve as markers for activity. The specific contributions of each investigation to the science themes and closely-linked L1 science objectives are as follows:

**Table 1:**
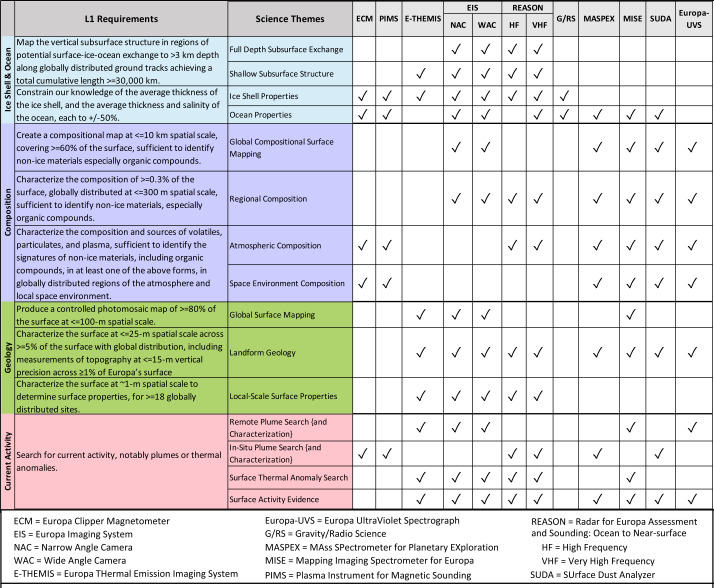
Linking of the three primary science objectives and cross-cutting Current Activity objective to the Level 1 science requirements, science themes, and contributing observations from the investigations. Achieving the high-level science objectives will enable the Europa Clipper mission to achieve its overarching goal of understanding Europa’s habitability

*Ice shell and ocean*: Data from EIS NAC and WAC along with the REASON VHF and HF ice-penetrating radar will contribute to investigating the potential subsurface exchange pathways in Europa’s ice shell and ocean; EIS NAC and WAC, REASON VHF and HF and E-THEMIS will interrogate the structure of Europa’s subsurface; ECM, EIS NAC and WAC, E-THEMIS, G/RS, PIMS, and REASON VHF and HF will constrain Europa’s ice shell properties, including its average thickness; and ECM, EIS NAC and WAC, G/RS, MASPEX, MISE, PIMS, REASON VHF and SUDA will constrain Europa’s ocean properties, including its average thickness and salinity.

*Composition*: Data from EIS NAC and WAC, Europa-UVS, MASPEX, MISE, and SUDA will enable construction of a compositional map comprising more than 60% of Europa’s surface at ≤10 km spatial scale; EIS NAC and WAC, Europa-UVS, MASPEX, MISE, REASON VHF and HF and SUDA will observe the composition of Europa’s surface at regions with ≤300 m spatial scale, including MISE observations at ≤50-m pixel scale of ≥30 surface features to determine composition of individual landforms; ECM Europa-UVS, MASPEX, MISE, PIMS, REASON VHF and HF, and SUDA will interrogate Europa’s atmospheric composition; and ECM, Europa-UVS, MASPEX, MISE, PIMS, and SUDA will measure the composition of Europa’s space environment.

*Geology*: EIS WAC and NAC images will enable production of controlled photomosaic maps covering ≥80% of the surface at ≤100 m spatial scale, with contributions by E-THEMIS and MISE observations at lower resolutions; EIS NAC and WAC, E-THEMIS, Europa-UVS, MASPEX, MISE, REASON VHF and HF, and SUDA will characterize landforms at ≤25-m spatial scale across ≥5% of the surface with global distribution, including measurements of topography at ≤15-m vertical precision across ≥1% of Europa’s surface; local scale surface properties will be observed by EIS NAC (∼1 m spatial scale) and WAC (≤4-m spatial scale), E-THEMIS (≤250-m), and REASON VHF reflectometry (≤10-km along track resolution) and HF reflectometry (≤27.5-km along track resolution) for more than 18 globally distributed sites.

*Current activity*: EIS NAC and WAC, E-THEMIS, Europa-UVS, and MISE will perform remote plume searches and characterize plume material if detected; ECM, MASPEX, PIMS, REASON VHF and HF, and SUDA will search for plumes through in situ measurements and characterize material if present; EIS NAC and WAC, E-THEMIS, MISE, and REASON VHF and HF will contribute to surface thermal anomaly searches to help define the source regions of potential plumes and activity; and EIS NAC and WAC, E-THEMIS, Europa-UVS, MASPEX, MISE, and REASON VHF and HF, and SUDA will contribute to searching for evidence of surface changes that indicate recent or current activity.

**Fig. 3:**
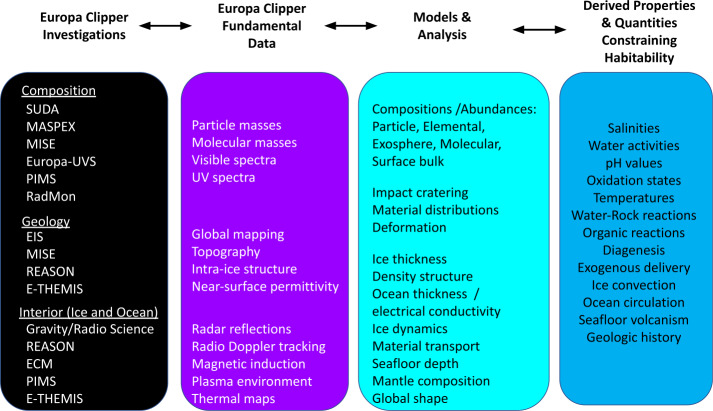
Europa Clipper investigations, left, will acquire the fundamental data—images, spectra, in situ samples, etc.—that will be interpreted through models and analyses to derive properties of Europa that reveal the types of metabolism that might be possible, and the environmental conditions that might support life’s origin and persistence through time. This synthesis of information is needed in order to assess Europa’s habitability

Constraining the compositional exchanges between suboceanic rocks, the ocean, the ice shell, and the surface throughout Europa’s history to understand its habitability provides an example for the need to combine the many lines of evidence inferred from the Europa Clipper investigations. Figure 3 describes pathways from the fundamental investigation data that will be obtained by Europa Clipper instruments, through data analysis and modeling, to a derived constraint on a parameter value important for habitability. Direct links are not made between measurements and derived properties because, in most cases, deriving the information requires data from multiple investigations.

To foster collaboration across all science investigations and mission development, the Europa Clipper mission has set up three Thematic Working Groups (TWGs): (1) Interior, (2) Composition, and (3) Geology. The charges of the TWGs map directly to the aforementioned high-level science objectives of characterizing the ice shell and ocean, composition, and geology. The TWGs are composed of science team members from all investigations and work to shepherd the respective discipline science across the multiple investigations (Pappalardo et al. 2024 this collection). In addition to the TWGs, Focus Groups (FGs) have been established to study specific topics that cross-cut the interest of the TWGs. For example, the current activity objective maps to the Plumes FG. Three additional FGs allow focused studies and discussion of radiation, geodesy science, and reconnaissance for future mission development.

At a level above the TWGs, the Europa Clipper science team structure also includes a Habitability Assessment Board (HAB), the membership of which is comprised of the entire Europa Clipper science team. The HAB is charged with providing a high-level, cross-instrument and cross-discipline, habitability-driven science perspective. Also, the HAB leadership is empowered to convene regular meetings to discuss and recommend to the project’s science leadership the formation of FGs to address scientific and technical problems related to habitability.

Together, these science team structures, science goals, and investigations enable the Europa Clipper mission to conduct its cross-discipline effort to assess Europa’s habitability. Measurements that feed into the higher-level objectives and cross-cutting topics are described in the following section.

## Measurements to Investigate Europa as a Potentially Habitable System

### Europa as a System

**Fig. 4:**
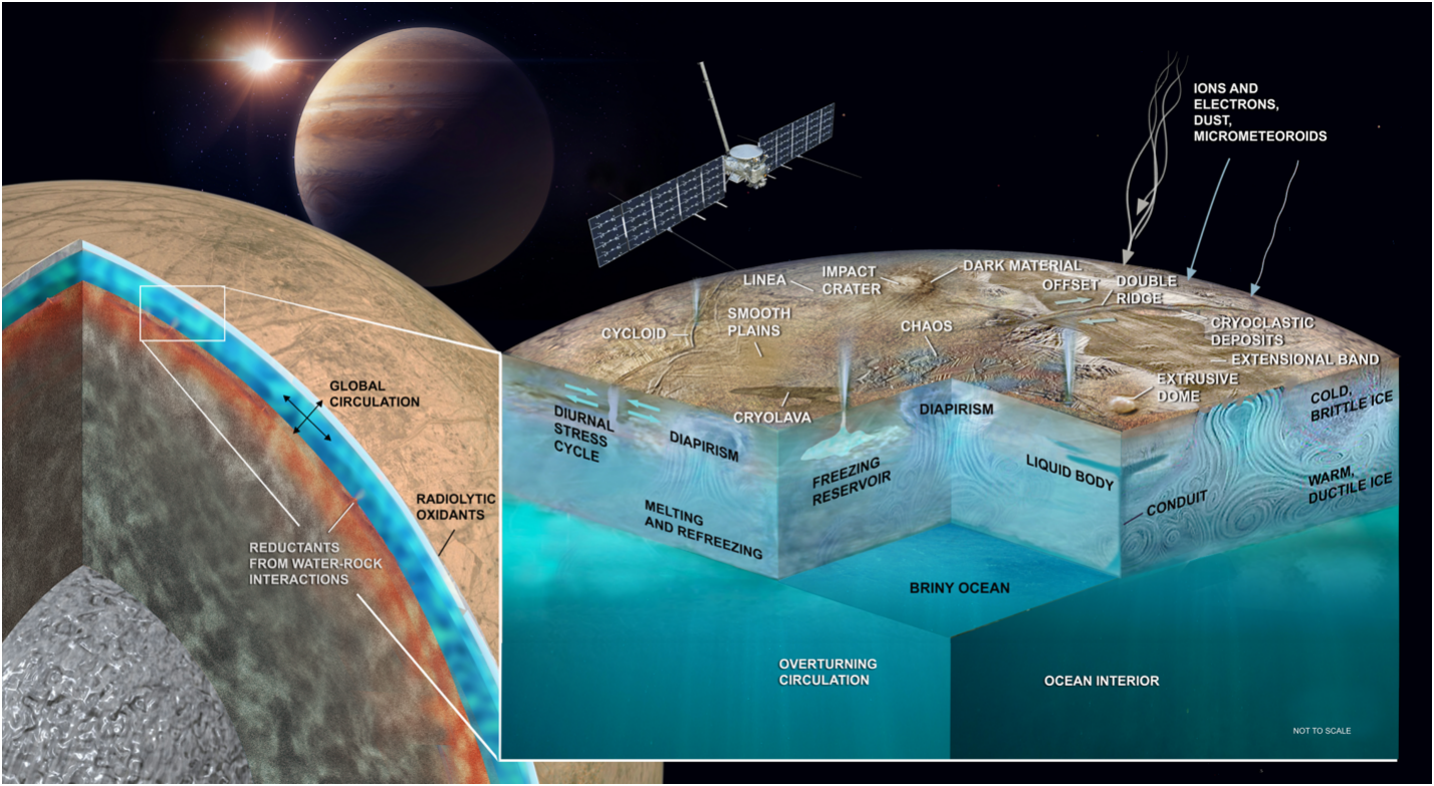
Europa is a global system with different interacting regions and processes. Europa Clipper will provide an unprecedented view into those processes, providing clues to the overturning of the ice and ocean, and the chemistry of the deeper interior. Artist credit: D. Hinkle

**Fig. 5:**
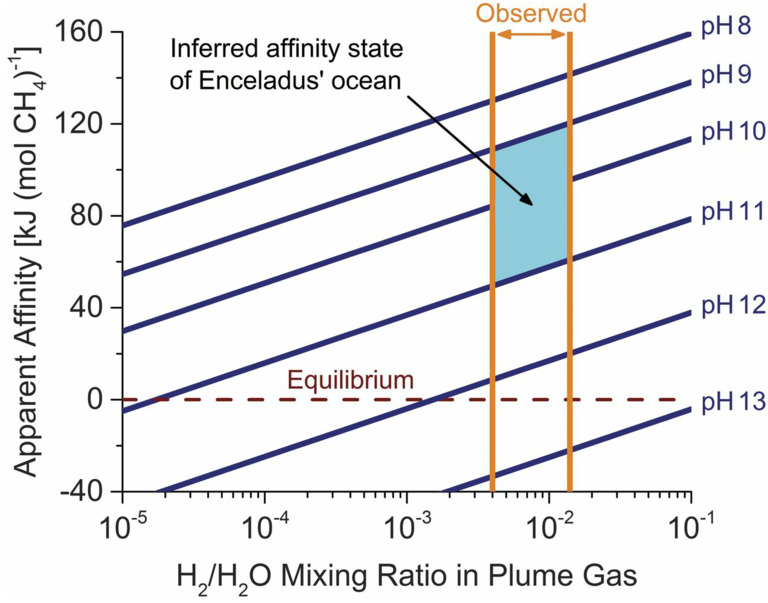
Chemical affinity for methanogenesis from CO_2_ and H_2_ inferred for Enceladus’s ocean, computed from available equilibrium thermodynamic data based on the volatile contents and particle-bound ions in south polar plumes. By similarly constraining the composition and volatile content of materials within Europa, either from plume materials that may be present, or from oceanic materials at the surface, the Europa Clipper mission may be able to constrain the chemical affinity available to support metabolic processes. From Waite et al. (2017). Reprinted with permission from AAAS

Investigating Europa’s habitability requires the synthesis of measurements that reveal the combination of material ingredients, energy sources, and geological processes capable of delivering these ingredients and energetic sources to viable ecological niches. Figure 4 combines themes from the associated Thematic Working Group papers (Composition: Becker et al. 2024; Geology: Daubar et al. 2024; Interior: Roberts et al. 2023, all this collection) to illustrate possible interior phenomena on Europa that may work together to create such potential niches. Understanding the parts of Europa and how they work together as a system requires understanding Europa’s formation (the initial inventory of energy and materials), physical processes that mix, reprocess, and redistribute these materials, and chemical pathways that arise from or feed back into those processes. Energetic pathways within Europa have been conceived, in global terms appropriate to the current state of knowledge, as fluxes of reductants and oxidants to the ocean over extended periods of time (McCollom 1999; Chyba and Phillips 2001; Zolotov and Shock 2003, 2004; Hand et al. 2007, 2009; Vance et al. 2016). These combinations of oxidants and reductants form a useful guide to the availability of chemical sources of energy for possible metabolisms in Europa’s ocean, ice shell, and suboceanic materials. If the Europa Clipper mission is able to infer the pH and composition of Europa’s ocean from measurements of the composition and volatile content of materials, either from any plume materials or from oceanic materials that may be present at the surface, the mission can provide the information needed to derive the chemical affinity—the excess energy available for a given metabolic reaction—in a similar manner as has been done at Enceladus based on Cassini observations (Waite et al. 2017; Glein et al. 2018). Figure 5, reproduced from Waite et al. (2017), depicts the putative bounds on the ocean’s pH, for the modeled escape mechanism of the measured H_2_ and H_2_O. The inferred excess chemical energy available for methanogenesis, shown on the y-axis, is highest for the lowest pH bound of 9.

**Fig. 6:**
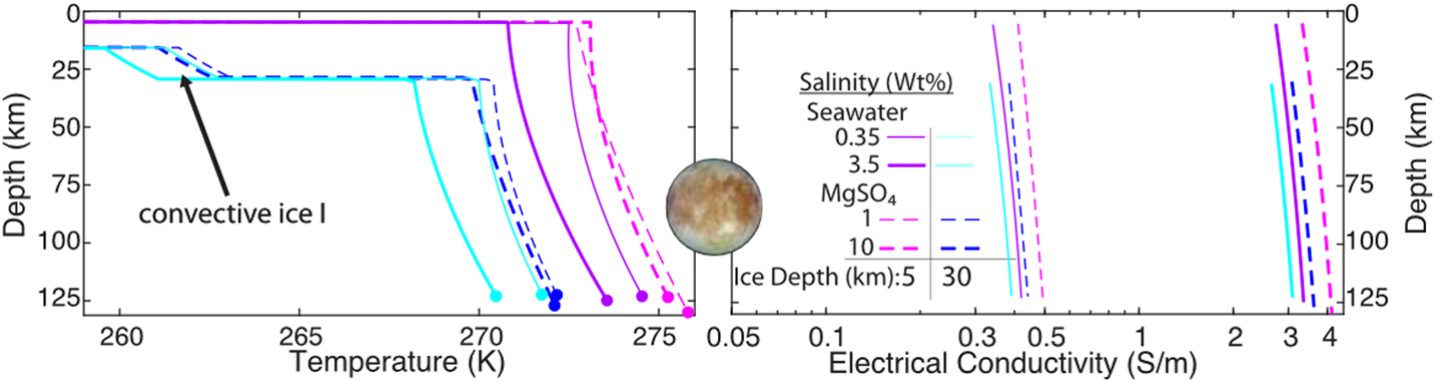
Europa’s ocean salinity is intrinsically linked to its temperature and to the thickness and dynamics of the overlying ice. For a given ice depth—5 and 30 km here—different compositions and amounts of salt require different melting temperatures of the ice, which sets the temperature at the top of the ocean. Convection in the ocean determines how temperature increases with depth as the salty fluids compress adiabatically. The temperature profile of the ocean strongly affects the conductivity of the ocean, which will be inferred from ECM and PIMS observations. Modified from Vance et al. (2021)

The availability of an energy source for life and presence of global liquid water are not sufficient conditions for habitability. An inventory of essential organic compounds; minerals; salts; dissolved volatiles; and associated pH, Eh, and water activity is a third requirement for the potential origin and persistence of life. The context of global and regional physical processes that drive chemical and energetic pathways also plays a role in creating and sustaining ecological niches by controlling the delivery of essential materials to different regions.


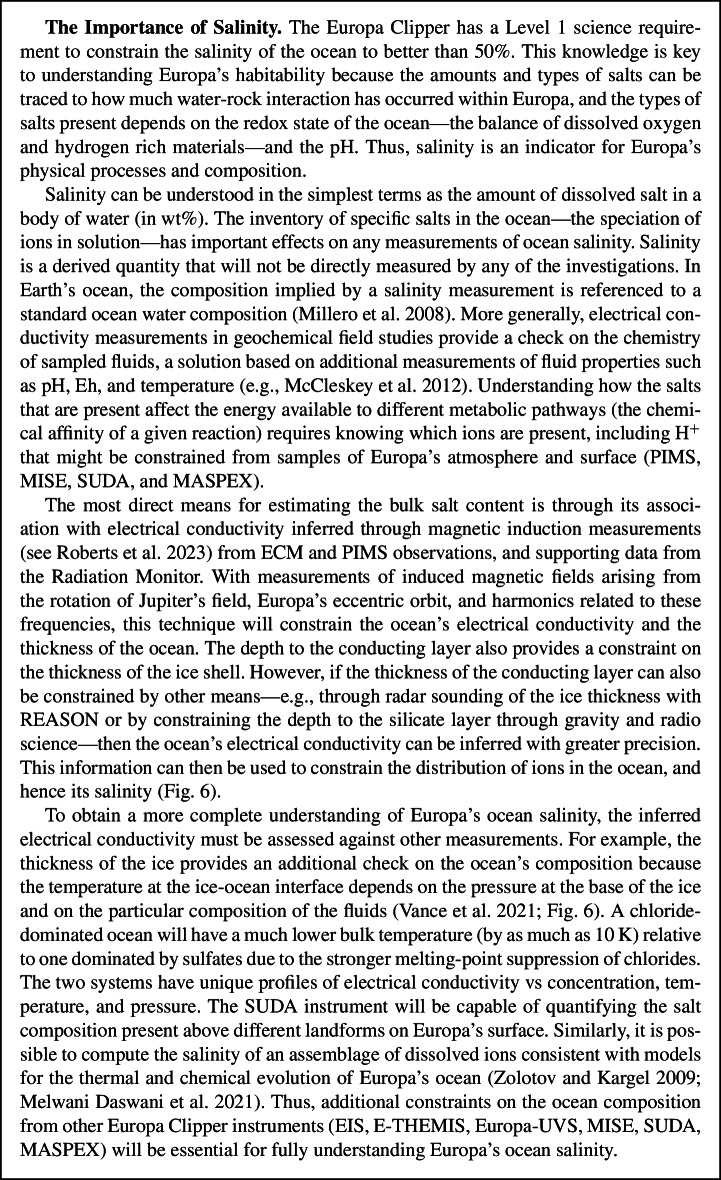


Europa Clipper will investigate Europa as a system by integrating data collected for the three primary and one cross-cutting science objectives as summarized in Table 1. Europa’s habitability is investigated by considering the many ways that these perspectives interact. As an example, consider that the apparent resurfacing of the majority of Europa’s ice shell over the last 100 million years (Bierhaus et al. 2009) indicates that there must be sources of energy to drive motion within the ice. Physical processes in the ice shell that create surface features (geology) redistribute material from the surface, ice shell, and ocean (interior, composition, and current activity) and are intimately tied to the planetary heat budget over time (interior). Thus, the vigor of these processes and the distribution and composition of features may reveal mechanisms of exchange between the surface, ice shell, and ocean that could introduce and sustain chemical gradients favorable to life or its precursors (habitability).

A systems science approach has also proven invaluable in the study of the Earth, both past and present. Such an interdisciplinary approach uses a holistic model that tracks the combined material and energy fluxes from individual subsystems to examine the stability, variability, and evolution of the larger system as a whole. Inferences about Earth’s biogeochemical evolution have been enabled by Earth systems science, leading to complex climate models (e.g., Kasting and Catling 2003; Edwards 2011; Catling and Kasting 2017), an extensive understanding of Earth’s evolution (e.g., Zeebe and Caldeira 2008; Ridgwell and Zeebe 2005; Lyons et al. 2014), and ocean models (e.g., Sarmiento and Toggweiler 1984; Fennel et al. 2005; Blättler et al. 2017). Similar methods have been employed for the study of Mars, leading to theories of its past conditions there (e.g., Hu et al. 2015) and hypotheses of possible metabolic activity (e.g., Formisano et al. 2004; Atreya et al. 2007; Lefèvre and Forget 2009).

**Fig. 7:**
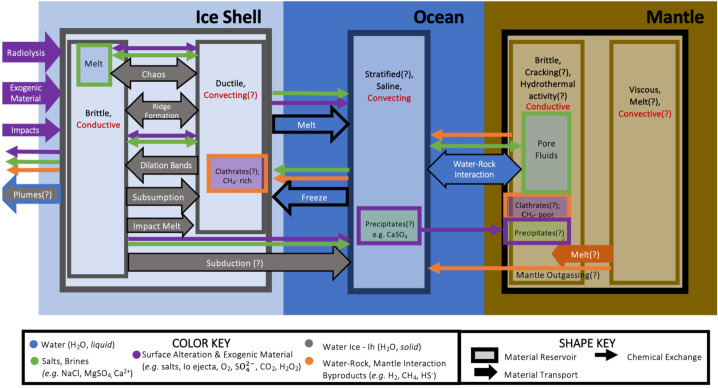
A systems model of Europa showing the interconnected systems of material exchange, thermal inputs, and reservoir state, building from the physical processes depicted in Fig. 4. Such a framework, common in Earth systems science, can be used to understand the overarching dynamics and stability of the system. It also illustrates how an input or perturbation to one part of the system may have wide-ranging impacts to other parts of the satellite system. Sizing of arrows and boxes is not quantitative

Using system science methods in the interpretation of Europa Clipper findings can lead to a better understanding of Europa’s formation, evolution, and current state. Figure 7 depicts different transport processes (arrows) and potential chemical reservoirs (boxes) in the Europa system, and their possible interactions. The remainder of this section describes aspects of Europa’s habitability in the context of this system framework, and further describes how the Europa Clipper mission will investigate them through the collection and integration of cross-cutting data.

### How Europa’s Formation and Evolution Determined the Distribution of Its Elements and Energy

By analyzing the compositions of endogenous and exogenous materials near Europa’s surface, the Europa Clipper mission will provide insight into the origin of materials from which Europa formed (Becker et al. 2024 this collection). Measurements of the moon’s composition by Europa Clipper will include remote compositional measurements, at global, regional, and local-scale resolutions, using infrared (MISE) and ultraviolet wavelengths (Europa-UVS), as well as making sounding and reflectometry observations to >3 km depth along globally distributed ground tracks achieving a total cumulative length ≥30,000 km (REASON). MISE will measure infrared absorption features indicative of hydrated salts, organics, water ice in various phases, and radiolytic products, and map compositionally diagnostic properties covering ≥60% of the surface at a global-scale of ≤10-km/pixel. These data will characterize the global-scale composition and chemistry and its large-scale variability; provide estimates of relative surface ages; identify exogenic compositional signatures; and enable the search for possible large-scale heterogeneity in the ocean at a regional-scale of ≤300-m/pixel for more than 50 surface features. These data will provide measurements of the regional-scale surface composition and chemistry, enabling a better understanding of the chemical pathways between the ocean and surface, implications for the habitability of the ocean, and will help identify any areas of current or recent activity. Observations at local-scale resolutions of ≤50-m/pixel for more than 30 surface features will determine the composition of individual landforms and investigate how composition influences the formation and evolution of landforms.

Europa-UVS will map compositionally diagnostic properties with ultraviolet observations across ≥70% of the surface at global-scale spatial resolution ≤30 km, in order to constrain the global-scale composition and chemistry. Observations at the regional-scale, ≤1 km/pixel, for more than 30 representative landforms distributed across the surface will constrain the surface composition and chemistry to understand the chemical pathways between the ocean and surface, provide implications for the habitability of the ocean, and enable identification of areas of current or recent activity.

REASON will make altimetry, near-surface reflectometry, and subsurface sounding measurements along globally distributed ground tracks to characterize the electromagnetic properties and interface geometries to depths of 3 km. Achieving a total cumulative length ≥30,000 km, in combination, these measurements will assess the regional composition of surface materials, the geological context of the surface, the potential for geological activity, the proximity of near-surface water, and the potential for active upwelling of ocean-derived material. These objectives will be achieved by ensuring measurements in regions that are globally distributed across the surface.

EIS NAC and WAC will obtain images at a global scale of ≤100-m/pixel (400-m/pixel in color) and at a regional scale of ≤25-m/pixel to understand the correlation of landform type with composition, study geologic processes relevant to habitability, and constrain surface ages. Imaging of limb profiles using EIS will help constrain ice shell properties, and topographic data derived from imaging observations will be used to characterize clutter in REASON sounding data.

Europa Clipper will also perform in situ measurements of sputtered and potential plume materials with ECM, PIMS, MASPEX, and SUDA to determine Europa’s composition and chemistry, including the identification of hydrated minerals and organic compounds. Volatile and particle measurements will map compositionally diagnostic properties of more than ten local surface features at a spatial resolution of ≤35 km in geographically distributed regions, with the resolution for the in situ (MASPEX and SUDA) measurements commensurate with spacecraft altitude. PIMS will measure the energy-per-charge distribution of ions in Europa’s ionosphere, possible plumes, and the magnetosphere to characterize their compositions and plasma sources and to constrain the radiation evolution of surface materials. ECM will measure the frequencies and amplitudes of ion cyclotron waves generated by pickup ions, which will be measured during wake crossings to determine the composition of Europa’s atmosphere.

Europa Clipper measurements will provide a detailed picture of Europa today. The challenge is to use these measurements to infer Europa’s accreted materials. Constraining, if not determining, these starting materials is key to understanding Europa’s chemical evolution and thus its habitability through time. Many sources for Europa’s bulk materials have been hypothesized: from local solar orbit (Canup and Ward 2002; McKinnon and Zolensky 2003; Mousis and Gautier 2004), more remote material (Ronnet et al. 2018), material reconstituted in an initially warm and relatively dense circumplanetary disk (CPD, Lunine and Stevenson 1982; Peale and Canup 2015; Moraes et al. 2018), or in the inner portion of the disk (Mosqueira and Estrada 2003; Batygin and Morbidelli 2020). Likewise, the extent to which the circumjovian thermal environment dictated the progression from rocky to ice–rock Galilean moons remains a subject of debate. Different primary drivers for the thermal gradient are possible. The temperature gradient of the circumplanetary disk may have induced partial devolatilization of the satellite’s building blocks (Canup and Ward 2002), whereas accretional heating may have led to hydrodynamic escape of a primary ocean (Bierson and Nimmo 2020), or both may have occurred. Alternatively, the ice-to-rock ratio in Europa could simply result from its accretion from hydrated carbonaceous chondritic materials that were heavily modified by subsequent radiogenic and tidal heating (Melwani Daswani et al. 2021; Mousis et al. 2023). The variety of different circumplanetary disk environments envisioned in different models is reviewed by McKinnon (2023). In any case, the amount and distribution of accretional heating depended sensitively on the size distribution of the satellitesimals (the satellite-building equivalent of planetesimals), the duration and intensity of accretional and subsequent bombardment, and the presence or absence of a primordial or accretionally-induced gaseous envelope around the growing bodies (e.g., Lunine and Stevenson 1982; McKinnon and Zolensky 2003; Bierson and Nimmo 2020).

By constraining the internal density and thermal state, Europa Clipper will provide clues to Europa’s origin and thermal evolution. Following an early phase of internal differentiation via separation of an ice shell from a rocky mantle that might have been partially hydrated, Europa’s long-term internal evolution was driven by long-lived radioisotope decay heat and tidal dissipation. These sources could drive thermal metamorphism of the rocky mantle (e.g., dehydration, decarboxylation) and partial melting of the silicates, leading to the separation of a metallic core (Greeley et al. 2004; Melwani Daswani et al. 2021; Trinh et al. 2023). The extent and nature of the latter is unknown, but refining Europa’s moment of inertia with the Europa Clipper G/RS experiment will help narrow down the range of possible internal structures. Europa Clipper will infer the internal mass distribution of Europa through determination of the moment of inertia by measuring the degree-2 static gravity coefficients with G/RS and the pole obliquity through analysis of G/RS data and EIS images. A knowledge of pole obliquity to 0.05 arcmin would be necessary to determine the polar moment of inertia in particular to an uncertainty of 0.004, which would be sufficient to constrain the interior structure (see Mazarico et al. 2023 this collection; Roberts et al. 2023 this collection), assuming that Europa is locked in a Cassini state, as is typical for synchronously rotating bodies (Baland et al. 2012).

Thermal metamorphism and magmatic activity could lead to the release of fluids and exposure of, e.g., basalts to the ocean and thus affect the ocean’s composition, redox, and pH during one or several episodes in the course of Europa’s history (e.g., Běhounková et al. 2021; Melwani Daswani et al. 2021). Measurements of gravitational tides by the G/RS experiment will recover the second-degree Love number, k_2_, at Europa’s orbital frequency with ≤0.06 absolute accuracy, which will confirm the existence or absence of a subsurface ocean. Furthermore, the ECM will characterize Europa’s magnetic induction response to variations of Jupiter’s magnetic field in the moon’s reference frame at two frequencies with an accuracy of $\leq \pm 1.5$ nT, which will constrain ocean and ice shell thicknesses and ocean conductivity. Additionally, Europa Clipper’s trajectory will provide the opportunity to deconvolve gravity anomalies arising from density variations in the ice shell from those due to seafloor topography. Among these, the seafloor topography will dominate the potential field anomalies even though Europa Clipper will be further than 2000 km from this location, enabling indications of sea floor conditions (Pauer et al. 2010; James 2016; Roberts et al. 2018).

### Energy from Tidal Heating

Along with neighboring moons Io and Ganymede, Europa is engaged in an orbital configuration called a Laplace resonance (e.g., Peale and Lee 2002). This resonance keeps the moons’ orbits slightly eccentric because the regular gravitational perturbations they exert on each other counteract the dissipation of tidal energy in their interiors, which serves to reduce their eccentricities. Tidal energy is contributed primarily by the varying gravitational force from Jupiter as the moons move nearer and farther from their parent planet throughout their eccentric orbits and as the position of Jupiter shifts back and forth with respect to Europa’s tidal axis. Europa Clipper will constrain the tidal energy input, as described further below.

Constraining the temporal evolution of Europa’s orbit contributes to understanding its current and long-term habitability. Europa has likely experienced enhanced tidal heating throughout much, if not all, of its history (Yoder and Peale 1981; Hussmann and Spohn 2004; Peale and Lee 2002). Also, due to their resonance, Io, Europa, and Ganymede maintain non-zero eccentricities that vary as angular momentum is continuously transferred outward (Lainey et al. 2009). For Europa, eccentricity could have varied by an order of magnitude over the satellite’s lifetime and respectively affected the heat production by tides (Hussmann and Spohn 2004; Sotin et al. 2009; Běhounková et al. 2021).

**Fig. 8:**
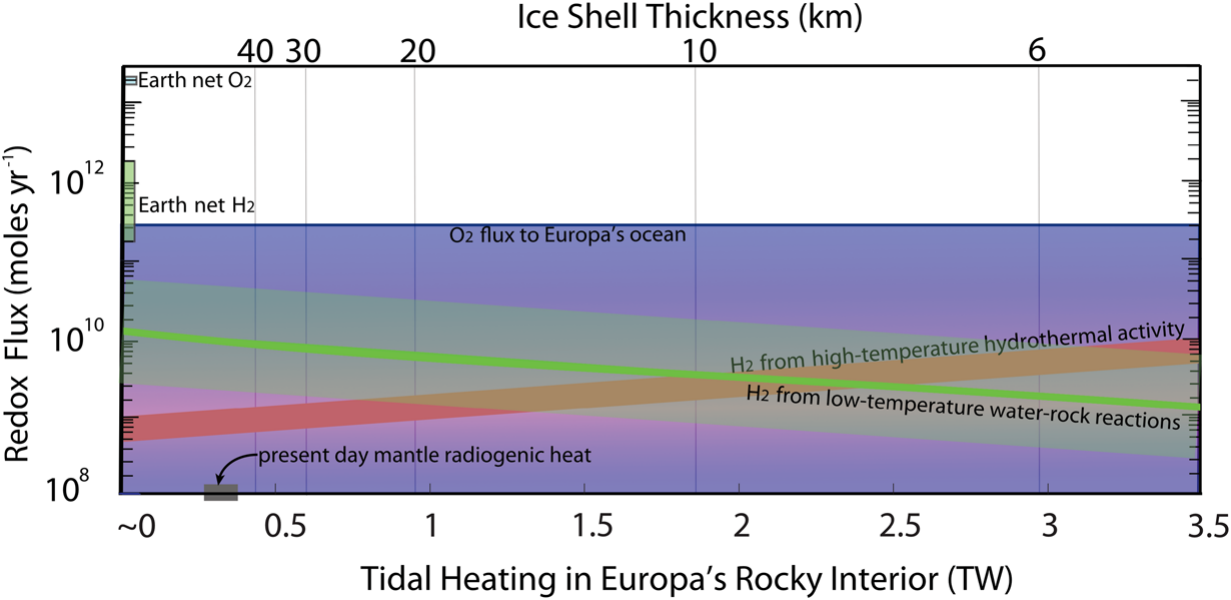
Europa Clipper may reveal the oxidation state of the ocean, which determines what kind of life might be able to exist there. Here, the modeled global fluxes O_2_ and H_2_ are inventoried on the y-axis, with the x-axis depicting the dependence of H_2_ flux on the extent of heating in the silicate layer. Less tidal heating enables deeper thermal fracturing and the potential for more H_2_ generated by water-rock chemistry (serpentinization). The bounds on O_2_ are broad, owing both to limited constraints on the magnitude and time-dependence of radiolysis of water at Europa’s surface, or of the efficiency of moving oxidants into the ocean. The balance of these reductant and oxidant fluxes determines the ocean’s pH and also the available energy for metabolism (e.g., Waite et al. 2017). Modified from Vance et al. (2016)

Precisely where in the interior of Europa the tidal energy is dissipated is a major open question. The ice shell may be the primary region where eccentricity tides give rise to a phase lag and thus to heating (Ojakangas and Stevenson 1989), but some dissipation also occurs in the core, mantle, and ocean (Sotin et al. 2009). At Europa’s orbital frequency and for plausible assumption with respect to ice rheology, tidal heat dissipation is expected to peak at a viscosity around 10^14^ Pa s, which is similar to the viscosity of water ice near the melting point. This heating could provide enough energy to maintain the subsurface ocean by creating melt (water) within the ice, which could drive exchanges of material and heat at the ice–ocean interface. Recent 3D modeling of tides suggests that tidal heating in the silicate interior may exceed the radiogenic heat, periodically providing enough heat to allow silicate melting and possibly drive seafloor volcanism (Běhounková et al. 2021). This melting in the silicate layer, along with melting in the ice, might enable the delivery of reductants and oxidants into the ocean, respectively (e.g., Schmidt et al. 2011; Vance et al. 2016; Steinbrugge et al. 2020; Hesse et al. 2021; Fig. 8).

To constrain tidal energy contributions, the Europa Clipper mission will collect measurements of gravitational tides with the G/RS investigation during the spacecraft’s close encounters with Europa, as noted above, to recover k_2_, and along with measurements of Europa’s degree-2 static gravity field, the mission will constrain Europa’s current internal structure (Gomez Casajus et al. 2021; Mazarico et al. 2023 this collection). In order to characterize the thermal state of the ice shell, these results will be combined with information about heat flow at the surface from direct measurements by E-THEMIS, which will map daytime and nighttime temperatures over ≥80% of the surface at a resolution of ≤25 km and with ≤2 K accuracy and precision, as well as passive radiometric measurements obtained during the Juno encounter of Europa in 2022 (Zhang et al. 2023). Inferences based on observations of geology (EIS, E-THEMIS, Europa-UVS, MASPEX, MISE, REASON, and SUDA), ice shell subsurface structure (ECM, EIS, E-THEMIS, G/RS, PIMS, and REASON), and composition (EIS, Europa-UVS, MASPEX, MISE, PIMS, REASON, and SUDA), the Europa Clipper mission will provide further insight into the sources of tidal heat in the interior. Also, in combination with long-term astrometry and data from the Juno and JUICE missions, a better understanding of the current migration rate of the satellites within the Laplace resonance can be obtained (Dirkx et al. 2017), leading to an understanding of how tidal heat might have changed through time.

### Elements for Life in Physical and Chemical Context: Is There Something to Eat?

The materials needed for life could have been accreted during Europa’s formation and produced over the history of the moon. They include compounds bearing sulfur, phosphorus, oxygen, nitrogen, carbon, and hydrogen (SPONCH). Europa Clipper’s measurements, described above, will return clues to the inventories of these materials and their products through compositional imaging and mass spectrometry, and by constraining the densities of the silicate layer and any metallic core.

The abundances and distribution of water, rocky materials, metals, and volatiles hold clues to Europa’s formation and evolution that will be revealed by Europa Clipper measurements. Based on studies of meteorites, refractory solids (rock, metals, organics; e.g., Zolotov and Kargel 2009) that were supplied to the CPD from which Europa accreted could have been both carbonaceous and non-carbonaceous. Contributions of very organic rich (cometary) materials from the outer solar system is possible as well, taking into account interpretations of gravity and composition data for Ganymede (Néri et al. 2020) and Ceres (Zolotov 2020). The solids that accreted to form Europa, including ices, were sourced from heliocentric solids, with a possible contribution to the more volatile solids from direct condensation in the CPD, depending on its temperature and pressure history (Prinn and Fegley 1989). As Jupiter grew toward its full mass, it opened a gap in the protosolar nebula, across which gas and coupled dust continued to flow, forming the CPD (e.g., Ayliffe and Bate 2009; Tanigawa et al. 2012; Szulágyi et al. 2016; Schulik et al. 2020), which in turn captured larger heliocentric solids (planetesimals and pebbles) (e.g., Ronnet and Johansen 2020; Batygin and Morbidelli 2020; Madeira et al. 2021; Cilibrasi et al. 2021). Solid materials thus, in principle, could have come from both inside and outside of this “Jovian gap” (Kruijer et al. 2017; Scott et al. 2018; Desch et al. 2018). As such, we predict that Europa will contain copious biologically essential elements such as oxygen, carbon, sulfur, and phosphorus, though not necessarily in solar amounts. As described in Sect. 3, Europa Clipper can investigate how these elements are processed in Europa’s interior by constraining their speciation and isotopic ratios, through measurements by MASPEX of the atmospheric and potential plume particles including: H_2_, H_2_O_2_, O_2_, Fe^2+^, Fe^3+^, CH_4_, CO, $\mathrm{CO}_{2}/\mathrm{HCO}_{3}^{-}/\mathrm{CO}_{3}^{2-}$, $\mathrm{NH}_{3}/\mathrm{NH}_{4}^{+}$, N_2_, $\mathrm{NO}_{2}^{-}$, $\mathrm{NO}_{3}^{-}$, $\mathrm{H}_{2}\mathrm{S}/\mathrm{HS}^{-}$, $\mathrm{HSO}_{4}^{-}/\mathrm{SO}_{4}^{2-}$, and through measurements by SUDA of Cl^−^, $\mathrm{SO}_{4}^{-}$, Na^+^, Mg^+^, and K^+^.

As a prime example, understanding the distribution of nitrogen and nitrogen-containing compounds will provide key constraints on Europa’s habitability because the isotopic composition and speciation of nitrogen provides clues to the oxidation state of the ocean. Europa Clipper will detect nitrogen and nitrogen-containing compounds through measurements by MASPEX of $\mathrm{NH}_{3}/\mathrm{NH}_{4}^{+}$, N_2_, $\mathrm{NO}_{2}^{-}$, $\mathrm{NO}_{3}^{-}$ and by Europa-UVS of N_2_. The provision and speciation of nitrogen depends on the amounts and chemistry of ice-forming solids accreted. Among major elements relevant to biogeochemical cycles in Europa, the global nitrogen budget is probably the least constrained at present (Zolotov and Kargel 2009). The largest source of nitrogen might be carbonaceous matter, both volatile and refractory (e.g., Miller et al. 2019). Direct incorporation of ammonia (NH_3_) and N_2_ might be less likely given that Europa’s ice-poor composition is generally ascribed to warmer conditions in the CPD closer to Jupiter and the evaporation of ices from pebbles that drift across the snow line towards Jupiter (e.g., Canup and Ward 2002; Ronnet et al. 2017). On the other hand, some formation models for the Galilean satellites have Europa analogs forming at greater (and colder) distances from proto-Jupiter, and migrating inward (e.g., Batygin and Morbidelli 2020). Accretional heating could also act to drive off volatile N_2_ and NH_3_, though ammonium minerals might remain stable in the interior (Mikhail and Sverjensky 2014). Even if initially depleted in nitrogen, there would be at least be some supplied by cometary bombardment over the history of the Solar System (Pierazzo and Chyba 2002), which might deliver ammonia in the form of ammonium salts (Altwegg et al. 2020).

Europa’s bulk inventory of carbon also depends on the retention of volatile CO_2_ or CH_4_ during and after formation. As mentioned, the MASPEX and Europa-UVS investigations on Europa Clipper will characterize compounds containing carbon, including CH_4_ and CO. Carbon may be retained in the rocky interior as graphite, bound organic materials, or sedimented organics or clathrates (Zolotov and Kargel 2009; Melwani Daswani et al. 2021). Sulfur from Io’s volcanoes is implanted on Europa’s surface (Carlson et al. 1999b; Trumbo et al. 2019). As in chondritic and cometary dust materials, sulfur is probably abundant in the rocky part of Europa, even if a large fraction has partitioned into a metallic core (Vance et al. 2014). The presence of sulfur and possible Mg-bearing compounds in endogenic surface materials (Becker et al. 2024 this collection) suggests sulfate-bearing oceanic water (Zolotov and Kargel 2009), though $\mathrm{SO}_{4}^{2-}$ cannot be the dominant anion in the ocean unless the ocean is relatively acidic. Even then, sulfate may be scavenged back into the rocky interior if volcanic hydrothermal systems persist (Tan et al. 2021). Phosphorus might be enriched at the seafloor due to such processing, but the limited solubility of phosphates (e.g., apatite) in ostensibly non-acidic water could hinder its availability in the ocean (Pasek and Greenberg 2012).

### Drivers of Energetic Disequilibria

Life on Earth gets its energy either through photosynthesis or by catalyzing favorable but sluggish chemical reactions through chemosynthesis. Relative to Earth, Europa’s greater distance from the Sun, harsh radiation conditions, and low temperatures render its surface much less likely to support photosynthesis in any familiar form. It follows then, that most discussions of how Europa may support life focus on chemosynthesis (e.g., Hand et al. 2009), which has the advantage of operating in the dark and over wide ranges of temperature and pressure—at least on Earth—and so may be relevant to Europa’s subsurface environments. Here we address the planetary processes that generate chemical energy sources that supports life as we know it, and the data from Europa Clipper that will be used to test whether such energy sources exist.

Geologically active planets can generate energetic disequilibria if their physical processes operate to mix materials faster than chemical reactions occur. The evidence for geological activity on Europa encourages speculation that mechanisms exist to sustain such disequilibria. Igneous and hydrothermal activity or rock fracturing at the ocean floor could provide sources of reductants (Vance et al. 2016; Běhounková et al. 2021), while radiolysis of water ice and sulfur species at the surface (due to Jupiter’s intense magnetospheric radiation) produces oxidants that could be transported into the ocean (Carlson et al. 1999a; Chyba and Phillips 2001; Kattenhorn and Prockter 2014). Interface environments where redox species first meet may serve as oases on Europa; examples include the ice–ocean interface (Russell et al. 2017) and hydrothermal vents (McCollom 1999). Alternatively, thermal energy alone can be translated to chemical energy. Reduced (e.g., H_2_) and oxidized (e.g., CO_2_) species can coexist stably at high temperatures, i.e., a finite H_2_ fugacity (the non-ideal equivalent of partial pressure) exists, but the mixture is unstable (out of thermodynamic equilibrium) if it is cooled rapidly enough so that the high temperature state is quenched (e.g., Shock et al. 2013). Hydrothermal fluids on Europa could support this process for compatible sets of temperature and fluid oxidation state (Hand et al. 2009).

Endogenous radiolysis of water is another means for generating chemical disequilibria (Bouquet et al. 2017). This phenomenon occurs where water molecules are in close proximity to radionuclides such as ^40^K and U/Th isotopes. Endogenous radiolysis is a single process that can produce both reduced and oxidized species. Thus, even geologically quiescent environments can continually (if slowly) generate sources of chemical energy. On Europa, relevant environments for radiolysis might include brines in the ice shell and the global ocean, if these fluids contain appreciable dissolved K, and rocks/sediments at the ocean floor if these solids are sufficiently porous to contain water (e.g., Ray et al. 2021). Estimates of porosity in Europa’s seafloor suggest that radiolytic production of hydrogen might extend to tens of km depth as compared with estimates of 5–10 km or more on Earth (see discussions in McKinnon and Zolensky (2003), Vance et al. (2007), Bouquet et al. (2017), and references therein). Measurements of K and ^40^Ar in Europa’s surface and atmosphere, respectively, can help to constrain the distribution of K in Europa’s ice, ocean, and interior, and the exchange paths between them. Europa Clipper can make measurements of these elements in the atmosphere or a potential plume with MASPEX (^40^Ar) and SUDA (K^+^), in any plume or cryovolcanic deposits with measurements by EIS of color center defects due to irradiation of salts including KCl (Poston et al. 2017; Hibbitts et al. 2019), or potentially, of chlorides by Europa-UVS (Trumbo et al. 2022).

Current estimates of the global fluxes of reductants and oxidants to Europa’s ocean—represented by the most common ones expected, H_2_ and O_2_—span a broad range that bears comparison to the global inventories for Earth (Fig. 8). High- and low-temperature water rock reactions might provide hydrogen rich materials in the range 10^8^ to 10^10^ mol/yr. An oxidant flux around 10^9^ mol/yr has been suggested based on the current understanding of oxidant implantation at the surface and the rate of overturning of the ice shell (Hand et al. 2007). The flux might be as high as 10^11^ mol/yr if the ice is saturated with oxidants (Greenberg 2010).

**Fig. 9:**
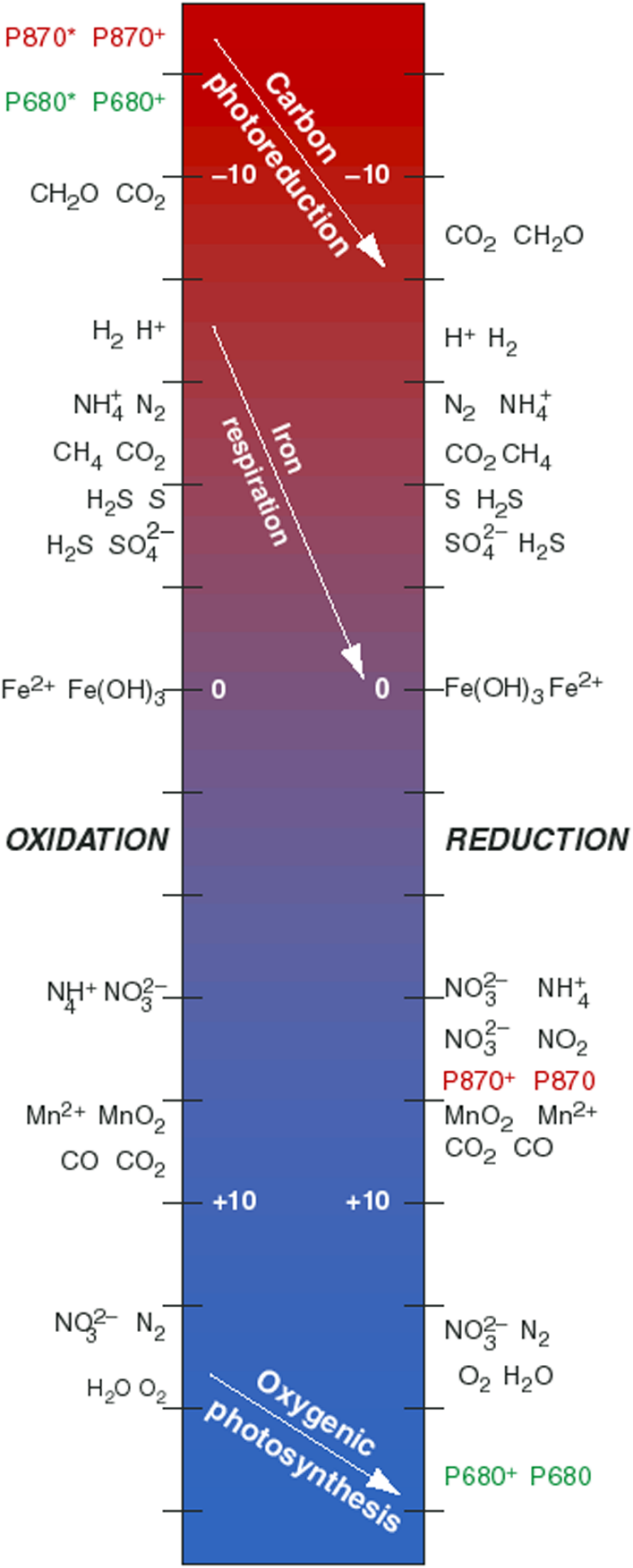
The relative energy available from different metabolic reactions can be constrained by the overall Europa Clipper investigation. The available energy is determined by the chemical disequilibria of materials provided by geological processes. In this figure, reactions of common inorganic substrates are plotted according to their redox potential at 1 atm, pH = 7, and 25 °C (i.e., neutral water at standard conditions). Only redox reaction pairs with a negative net redox potential can provide energy; oxygenic photosynthesis, as on Earth, requires energy (sunlight). The arrows indicate three metabolic reactions, two of which are photosynthetic processes facilitated by complex biomolecules that are not known to exist on Europa. By gathering an inventory of the simpler inorganic materials and constraining their fluxes in Europa’s ocean, ice, and seafloor, the Europa Clipper mission can assess the pathways that might be available for life. More complex biomolecules involved in metabolism, such as the photosystem complexes indicated here (P870 and P680), if detected, might be interpreted as biosignatures if the simpler substrates they mediate were also found. From Gaidos et al. (1999). Reproduced with permission from AAAS

The synthesis of multiple Europa Clipper measurements will be needed in order to reveal sources of energy for life. Direct observations that yield abundances of solid and volatile compounds (measurements by MISE, MASPEX, SUDA, and Europa-UVS as described in Sect. 2.2 & 2.4 and shown in Table 1) can be interpreted using models of exogenous delivery, and escape from sputtering and sublimation, to assess the extent to which reactions among those compounds have equilibrated. An example of the synthesis approach is provided by the assessment of energy availability from methanogenesis at Enceladus using data collected by Cassini from that satellite’s south polar plume (Waite et al. 2017). For such an investigation it is critical to measure many species with different oxidation states—e.g., H_2_, H_2_O_2_, O_2_, Fe^2+^, Fe^3+^, CH_4_, CO, $\mathrm{CO}_{2}/_{3}^{-}/\mathrm{CO}_{3}^{2-}$, $\mathrm{NH}_{3}/\mathrm{NH}_{4}^{+}$, N_2_, $\mathrm{NO}_{2}^{-}$, $\mathrm{NO}_{3}^{-}$, H_2_S/HS^−^, $\mathrm{HSO}_{4}^{-}/\mathrm{SO}_{4}^{2-}$—so that the richness of reductant–oxidant combinations can be assessed as completely as possible in terms of their ability to provide sufficient energy for life (Fig. 9; e.g., Gaidos et al. 1999; Hand et al. 2009).

On Earth, organic compounds bearing signatures of their source regions and conditions are transported from subsurface environments that are difficult to access directly (Shock et al. 2019; Sephton et al. 2018). Relative abundances of organic compounds compared to thermodynamic models serve as tracers of subsurface temperature and redox states within various hydrothermal systems (Shock 1988, 1989, 1994; Helgeson et al. 1993; Cruse and Seewald 2006; Proskurowski et al. 2006; Tassi et al. 2007; Lang et al. 2010; Shock et al. 2013; McDermott et al. 2015). Similar techniques could be used to constrain pressure, pH, and other compositional variables for subsurface environments that release organic compounds to more accessible sampling locations (Robinson et al. 2021).

Europa Clipper’s two mass spectrometers will enable characterization of geochemical processes operating beneath the surface by sampling materials lofted to the altitude of the spacecraft either by processes occurring on the surface or through plume activity (if present). MASPEX can identify and quantify volatile organic compounds with a large variety of chemical functional groups, as described in Sect. 2.4 (Brockwell et al. 2016; Waite et al. 2024 this collection). SUDA expands this capability by measuring the composition of exospheric grain particles (ice or non-ice) and can detect organic compounds, salts, and minerals (Kempf et al. 2014, 2024 this collection). Also, analog studies impacting ice grains at hypervelocities of ∼5 km/s on targets simulating the Europa Clipper conditions for SUDA have obtained quantitative detection limits for a wide range of amino acids and fatty acids mixed in liquid water (Klenner et al. 2020). Both MASPEX and SUDA possess much improved sensitivity and mass resolution (see Kempf et al. 2024 this collection; Waite et al. 2024 this collection) compared to their heritage instruments built for the Cassini mission, which identified a diverse suite of organic compounds in the plume of Enceladus (Postberg et al. 2018a,b; Khawaja et al. 2019). MASPEX will characterize Europa’s atmosphere sufficient to identify the global distribution of major volatiles and resolve key organic compounds, their sources, and their relative abundances where volume mixing ratios exceed $1.7 \times 10^{-4}$. The organic inventory detectable at Europa’s surface—either by mass spectrometers or spectral imagers—may provide a similarly rich data set that can be more comprehensively characterized and used to constrain the geochemical processes and fluxes of materials into/out from Europa’s subsurface. For example, the Cassini Visual and Infrared Mapping Spectrometer (VIMS) mapped cycloalkanes, olefinic compounds, CH_3_OH, and polycyclic aromatic hydrocarbons on Iapetus. The hemispheric asymmetry of these materials plausibly indicated deposition of pristine organic-rich dusts from the region beyond Neptune where Phoebe presumably originated (Cruikshank et al. 2014). Putting all of this information together with the physical context of material cycling will provide a picture of allowed metabolic processes allowed by inferred redox potentials, such as those pictured in Fig. 9. In this illustration, two photosynthetic processes used by complex eukaryotes are pictured. While photosynthesis and eukaryotes are not predicted at Europa, these metabolic processes demonstrate how complex molecules (P870 and P680) that facilitate metabolism might serve as biosignatures if found in the context of appropriate redox potentials.

### Habitability Through Time

**Fig. 10:**
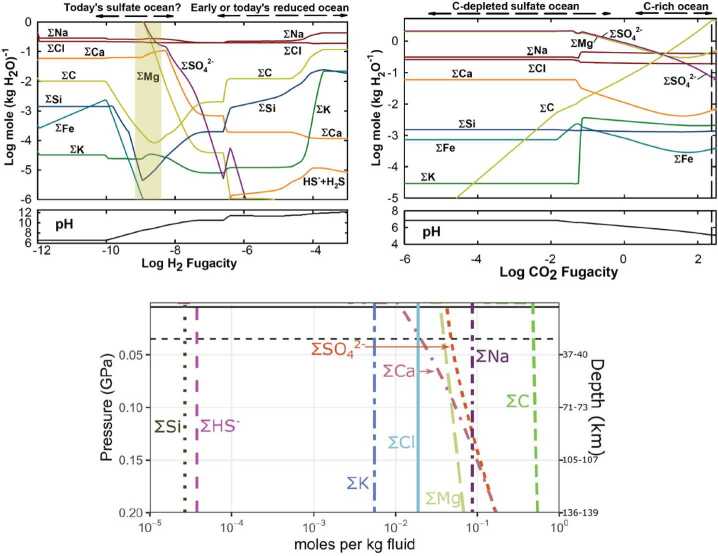
The composition of Europa’s ocean is a record of how Europa evolved. In some scenarios, an initially H_2_-rich ocean would have become more oxidizing over time due to the influence of radiolytically produced oxidants from the surface and/or H_2_ escape to space. Some models (upper left; modified from Zolotov 2008; T = 0 °C, P = 0.1375 GPa, water/rock ratio (W/R) = 1 by mass, 10% of reacted C in carbonaceous chondrites) predict that decreasing $f\mathrm{H}_{2}$ might have driven the ocean composition from one dominated by chlorides to one dominated by sulfates (for log $f \mathrm{H}_{2}<-9$). Alternatively (upper right; also from Zolotov 2008; T = 0 °C, P = 0.1375 GPa, W/R = 1, log $f \mathrm{H}_{2}=-10$), if the ocean retained significant abundances of inorganic carbon species, concentrations of sulfate ions are compatible with those of chlorides. Another model (bottom; from Melwani Daswani et al. 2021. Adopted with permission from John Wiley and Sons.), that also considered the retention of CO_2_ (here, nominal pH of 5.5, in equilibrium with a CM chondrite composition) shows the effects of varying solubility of ions with depth. Differences between the ion abundances are attributed to different input mineral compositions

The Europa Clipper mission may provide information on how energy sources and chemical inventories have changed through time, reflecting changes in parameters that affect habitability. Geologically slower changes in parameters affecting Europa’s habitability might cause adaptation of possible organisms, similar to life evolving to adapt to changing conditions on Earth (e.g., Dietrich et al. 2006). In turn, these changes stem from the geochemical evolution of Europa’s interior, affected by its thermal and orbital evolution (e.g., Zolotov and Kargel 2009; Hand et al. 2009; Vance et al. 2016; Běhounková et al. 2021; Melwani Daswani et al. 2021; Fig. 10). On geological timescales of millions of years, global habitability is affected by changes in the intensity and distribution of tidal dissipation and radiogenic heating, which in turn affect the thicknesses and dynamics of the ice shell and the ocean layer, potential magmatic and hydrothermal activity in the vicinity of oceanic floor, the ocean’s composition, and chemical sources of energy for metabolism.

On the time scale of the current surface age (∼100 Myr; Bierhaus et al. 2009), the ocean’s pH might have changed due to increasing salinity as the ice thickened; that is, if it thickened, which is the leading geological interpretation at present (e.g., Greeley et al. 2004). The changing ocean composition would have influenced the potential for metabolic reactions (Hand et al. 2009). In deeper, possibly convecting parts of the icy shell, changing temperature, pressure, and brine content affect habitability at a local scale. Rapid geologic changes affecting habitability might have been caused by infrequent large impacts (Zahnle et al. 2003) that penetrated (Cox and Bauer 2015) or partially exposed the deeper icy shell (Carnahan et al. 2022), and by cooling and tidally driven fracturing of the shell causing upwelling of oceanic water forming dikes and possibly surface ridges (Craft et al. 2016; Buffo et al. 2020). In both of these example processes, low-pressure water boiling leads to degassing and freezing that affect the pH, salinity, and composition of oceanic water. Fast changes might also occur from the formation of lenses or other bodies of water within the shell, followed by freezing (Schmidt et al. 2011; Manga and Michaut 2017; Chivers et al. 2021). Europa Clipper’s high-resolution imaging by the EIS investigation will reveal the details of such possible cryovolcanic processes, and EIS global mapping will improve our understanding of the influence of impacts (see Turtle et al. and Daubar et al. 2024 this collection). Compositional measurements (MISE, Europa-UVS, SUDA, MASPEX) and radar sounding by REASON covering overlapping spatial regions will make it possible to infer the chemical consequences of such cataclysms.

An episode of flood silicate or non-silicate (e.g., sulfurous or organic) volcanism at the ocean floor would affect physical and chemical processes at the ocean floor, oceanic acid–base and redox conditions, organic and inorganic inventories, and chemical sources of energy for metabolism. The Europa Clipper G/RS investigation will make observations of gravity anomalies arising from density variations within Europa, as well as detect line-of-sight residuals from local to regional scale features within the ice shell and on the seafloor. Comparison to geologic features, near-surface radar detections from the shell, and crossovers sensing the same gravity anomalies but from different altitudes could help distinguish anomalies arising from the shell from those at the sea floor. In particular, the sizes of the seafloor anomalies might be used to constrain the thermal state of the silicate interior, with implications for the habitability of the sea floor (Pauer et al. 2010; James 2016; Roberts et al. 2018; Koh et al. 2022; Mazarico et al. 2023 this collection).

Europa’s icy surface contains the most accessible record of clues to any changes in its habitability over the past ∼100 Myr. Stratigraphic analyses based on Galileo imaging data indicate that extensive ridge plains are the oldest features, whereas chaos terrains appeared most recently (Figueredo and Greeley 2004; Leonard et al. 2018). Geological mapping based on imaging (EIS, E-THEMIS), coupled with G/RS gravity measurements and REASON radar observations of the subsurface density contrasts in the ice shell, which can indicate impurities (salts, brines) and/or liquid water pockets, is expected to yield a more comprehensive assessment of the timeline of geological processes and their mechanisms (Daubar et al. 2024 this collection). For example, whether chaotic terrains and potential brine rich intra-ice melts are relatively recent, or occurred for much of Europa’s geologic past, may be determined through REASON radar observations searching for liquid water lenses or layers of salts formed as these lenses freeze out (Chivers et al. 2021). Observations by MISE, EIS, Europa-UVS, SUDA, and MASPEX are expected to reveal the associated compositions of the surface materials, including the diagenesis due to internal melt fractionation and subsequent alteration by radiation and micrometeorites.

Temporal changes in the composition of surface endogenic materials (e.g., salts) should inform about compositional changes in the icy shell and/or in the ocean. The resulting assessment of exogenic and radiolytic material delivery into the subsurface as a source of acids, sulfates, and strong oxidants (O_2_, H_2_O_2_, etc.) would constrain the potential oxidant flux to the ice shell and ocean (Hand et al. 2007). MASPEX data on ^40^Ar and volatile elements in plume gases, if present, and SUDA measurements of potential plume particles, of salts, organic compounds, and minerals would constrain the timing and scale of chemical fluxes in the mantle–ocean–ice shell system. The assessments of past ocean composition can be benchmarked by Europa Clipper investigations with evaluations of the oceanic composition through sampling of plumes (if present) and fresh surface deposits, and from the present-day electrical conductivity of the ocean (Becker et al. 2024 this collection).

## Potentially Habitable Environments Within Europa and How Europa Clipper Will Study Them

### Within the Ice

To contain a habitable environment in its ice shell (Fig. 4, middle right), Europa must transport surface materials (that contain radiologically produced oxidants) downward into the ice shell and also upwards from within the ocean (with theorized hydrothermally produced reductants; Fig. 4, left, and lower right). Europa’s surface records many distinct resurfacing processes that occurred over the last 100 Myr (Bierhaus et al. 2009) that may have enabled surface-subsurface mixing. This young surface age suggests that the ice may have been active and constantly resurfacing over its lifetime, albeit with evolving geological styles (Doggett et al. 2009). Alternatively, a catastrophic event, causing overturning of the ice shell may have occurred sometime around 100 Myr ago (Pappalardo et al. 1999), implying a large flux of oxidized materials into the ocean. If overturning is regional or more sporadic, involving surface materials much older than 100 Myr (e.g., Bierhaus et al. 2009; Howell and Pappalardo 2019), the delivery of materials into the ocean may be more limited (e.g., Russell et al. 2017). Observations of the geology, composition, and searches for current activity across the Europa Clipper investigations will provide further constraints on the surface age on the global- (∼100 s m pixel scale) down to local scales (<1 m pixel scale).

Activity within the ice shell will also contribute to the potential for habitable environments there. The thickness of Europa’s ice shell both results from, and is a function of, its thermal structure (e.g., McKinnon 1999; Barr and Showman 2009). An imbalance between heat generation and transport can result in changes to the thickness of the ice shell, while the onset or slowing of solid-state convection can also modulate the ice’s thickness (e.g., Mitri and Showman 2005). Convection may also promote material exchange across the ice shell (e.g., Allu Peddinti and McNamara 2015). If the thickness of Europa’s ice shell is greater than 10 km, as suggested by modeling (e.g., Pappalardo et al. 1998; Quick and Marsh 2015; Howell 2021), the interior of the ice may be undergoing solid-state convection (McKinnon 1999; Sotin et al. 2002; Howell et al. 2021). In this case, stress and rheological conditions in the ice shell may inhibit transport of water from the ocean directly to the surface (e.g., Crawford and Stevenson 1988; Manga and Wang 2007; Kattenhorn and Prockter 2014), although many inter-shell transport processes have been hypothesized (Daubar et al. 2024 this collection).

Dissolved ions from the ocean have been assumed to enter the bottom 10% of the ice (e.g., Marion et al. 2003, 2005; Vance et al. 2019; Soderlund 2019; Soderlund et al. 2020) and any freezing of the ocean could entrain oceanic materials into the ice (Soderlund et al. 2014; Buffo et al. 2020, 2021; Wolfenbarger et al. 2022a). This deeper ice shell or oceanic material may be delivered upwards, to shallower depths and possibly to Europa’s surface through the formation of band structures (e.g., Howell and Pappalardo 2019), fluid-filled fractures (e.g., Craft et al. 2016; Buffo et al. 2020), chaos terrains (e.g., Sotin and Tobie 2004; Schmidt et al. 2011), and/or injected sills (e.g., Michaut and Manga 2014; Chivers et al. 2021; Culberg et al. 2022). Effusive eruptions to the surface may also occur when excess pressures, created by the gradual freezing of mid-shell liquid reservoirs, or by tidal stresses acting on these reservoirs create fractures through which fluid is subsequently pushed to the surface (Pappalardo et al. 1999; Fagents et al. 2000; Fagents 2003; Sparks et al. 2017; Quick et al. 2017, 2022; Lesage et al. 2020a,b; Steinbrugge et al. 2020; Chivers et al. 2021). For downward transport of material, geological processes in the ice that may entrain oxidants into the interior include: in situ melting of the brittle lid (e.g., Schmidt et al. 2011); subsumption of the crust (Kattenhorn and Prockter 2014); deep impact melting (Carnahan et al. 2022); and brine percolation delivering shallow brines (Kalousová et al. 2014) or surficial oxidants (Hesse et al. 2021). Europa Clipper investigations, namely EIS, REASON, ECM, PIMS, G/RS, MASPEX, MISE, SUDA, and E-THEMIS will further characterize the structure of Europa’s ice shell across at least 60% of the body, providing indications of overturn and/or transport processes that could enable habitable subsurface environments in the ice shell and/or ocean.

Material exchange within Europa’s ice shell could produce reservoirs of liquid water with the conditions for supporting life. Owing to their elevated temperatures relative to the surrounding ice, rising diapirs may create habitable melt regions in the ice shell as they intrude into the brittle lithosphere, warming it above one or more eutectic temperatures (depending on the salts present in the shell), and possibly even merging to create warm chaos regions (Ruiz et al. 2007; Schmidt et al. 2011; Michaut and Manga 2014; Singer et al. 2021). Near-surface liquid reservoirs might also be generated through the injection of water from the ocean below as dikes (e.g., Craft et al. 2016; Buffo et al. 2020) or sills (e.g., Michaut and Manga 2014; Chivers et al. 2021; Culberg et al. 2022). The thermal evolution of such reservoirs would control their longevity. If these reservoirs contain freezing point depressants such as salts or mineral acids (Bonales et al. 2013; Muñoz-Iglesias et al. 2013; Quick and Marsh 2016; Lesage et al. 2020a,b, 2021), they could remain liquid for >10^3^ years (Schmidt et al. 2011; Quick et al. 2022; Chivers et al. 2021) and might therefore become perched habitable regions within the ice. As specified in Table 1, REASON, E-THEMIS, and EIS will contribute to “mapping the vertical subsurface structure in regions of potential surface-ice-ocean exchange to >3 km depth along globally distributed ground tracks,” and so will improve our understanding if these liquid pockets exist and if they may indeed be formed and supported by the conditions that would make them habitable.

Smaller-scale, potentially habitable regions include mL-volume brine pockets akin to those found in terrestrial sea ice (Boetius et al. 2015; Deming et al. 2017; Buffo et al. 2020, 2021; Wolfenbarger et al. 2022b) or in temperate meteoric (glacial) ice, μL-volume liquid inclusions between grains, or in liquid films at the surfaces of entrained mineral grains (Priscu and Christner 2004; Price 2007). On Earth, these habitats may be able to support life on 100,000-year timescales (Price 2000). Such brine pockets in Europa’s ice might also provide chemical energy and nutrients, just as in terrestrial subglacial habitats where sunlight cannot penetrate the ice (Boetius et al. 2015). Near the ice–ocean interface, nutrient exchange in the ice is coupled to the brine volume, which governs the permeability of ice (Arrigo 2014). Also, platelet ice on Earth is thought to exchange interstitial seawater during tidal cycles (Arrigo et al. 1995). On this basis, regions of the ice shell where more rapidly accreted frazil ice—loose, randomly oriented, plate or discoid ice crystals formed in supercooled turbulent water—may have more favorable habitats than those where slower accreting congelation ice forms (Wolfenbarger et al. 2022a). Europa Clipper will employ radar probing of the subsurface with the REASON investigation to identify brine in the ice. The presence of brine could manifest as reflections from the subsurface (if detectable) or highly attenuating regions in the ice (Blankenship et al. 2024 this collection). Surface imaging and compositional measurements by EIS, Europa-UVS, MISE, MASPEX, and SUDA can then place these highly radar-attenuating regions in the context of thermal anomalies, chaotic terrains, compositional markers, and/or other geologic features consistent with melting (e.g., pits, domes and micro-chaos; Singer et al. 2021). The complex dielectric responses of intra-ice brines should provide clues to their composition that are important for understanding the habitability of brine pockets.

The composition of water within the ice shell will determine whether life might be viable in pockets of fluid within the ice. The salinity, size, and composition of brine pockets evolves as temperature decreases (e.g., Wolfenbarger et al. 2022b). As liquid progressively freezes, rejecting salts from the ice and concentrating them in the residual brine (as on Earth, e.g., Petrich and Eicken 2017), salt minerals will start to precipitate, altering (fractionating) the composition of the brine (i.e., the relative concentration of dissolved ions) and the precipitating salts (Zolotov and Shock 2001, 2004; Marion et al. 2005; Vance et al. 2019; Wolfenbarger et al. 2022b; Chivers et al. 2021). These effects likely cause a gradient in brine chemistry from the ice–ocean interface to the ice shell interior. Near the surface, material exchange might occur via entrainment of both endogenous salts and acids formed or altered by radiolysis (Carlson et al. 2009; Hand et al. 2009) and silicate materials and salts implanted or derived from Io (Postberg et al. 2006; Alvarellos et al. 2008). Nutrient delivery into the ice from the ocean might occur where the ice shell is permeable and tides drive fluids into and out of the ice, or depend on melting and freezing cycles. Based on studies of sea ice, the permeability of Europa’s ice might be negligible below a critical porosity of 5% (Golden et al. 1998, 2007). As shown in Table 1, REASON, E-THEMIS, and EIS will contribute to “mapping the vertical subsurface structure in regions of potential surface-ice-ocean exchange to >3 km depth along globally distributed ground tracks” to inform us on whether these potentially habitable water pockets exist and what conditions they create.

Previous measurements of surface composition have shown that Europa’s icy surface contains salts, acids, and hydrogen peroxide (McCord et al. 1998a,b; Carlson et al. 1999a,b), which might suggest limited habitability in regions where salt becomes too concentrated. A brine that becomes progressively enriched in divalent cations (e.g., Mg^2+^) as it freezes will become increasingly chaotropic—disruptive to hydrogen bonds—and thus destabilizing to life within these potential habitats (Hallsworth et al. 2007; Oren 2013). When multiply charged divalent or multivalent ions dominate, such high ionic concentration is a challenge to the habitability of the environment, even in the presence of otherwise sufficient water activity (Fox-Powell et al. 2016). In the case of monovalent salts (e.g., NaCl), on the other hand, even saturated environments have been found to be moderately habitable (Lee et al. 2018). Europa Clipper will make observations of surface composition, including salts, to shed light on the materials’ origins and processing to constrain what may exist at the subsurface. As described in Table 1, EIS NAC and WAC, Europa-UVS, MASPEX, MISE, and SUDA will make a compositional map of more than 60% of Europa’s surface at ≤10 km spatial scale and EIS NAC and WAC, Europa-UVS, MASPEX, MISE, REASON VHF and HF and SUDA will collectively observe the composition of Europa’s surface at selective regions at a finer (≤300 m) spatial scale to identify non-ice materials.

Life on Earth shows a robust ability to adapt to saline environments—limits on the habitability of hypersaline brine environments continue to be challenged by the discovery of life in environments which are otherwise deemed uninhabitable (e.g., Cubillos et al. 2019). For instance, within sea ice environments, many microbes produce and exude molecules that either reduce brine drainage from the pore spaces, increasing the pore space salinity (Krembs et al. 2011), or help said microbes to adhere to surfaces and survive adverse conditions (Poli et al. 2010). Cold, saline conditions in sea ice pores impose selection pressures that cause long-lived, isolated communities within the pores to specialize and reduce their biodiversity, even while those communities become more biologically productive (Cooper et al. 2019). Similar processes may operate in pore fluids within Europa’s ice shell. Therefore, while measurements by Europa Clipper investigations will possibly detect whether water pockets exist within the ice shell and place constraints on their thermal conditions and compositional make-up (see previous paragraphs describing investigation measurement details), the habitability of those environments will only be constrained by our current understanding of life as we know it and the likelihood that life may be capable of survival there.

### Within the Ocean

The habitability of Europa’s ocean is determined by its chemical and physical evolution through time. The composition of oceanic water affects the pH, redox state, redox disequilibria, water activity, ionic strength, and some physical properties of the ocean. In turn, the ocean composition holds clues about the ocean’s origin and evolution, ongoing exchange processes in the ice–ocean–rock/sediment system, and chemical reactions in the ocean and at the water–rock interface. The ocean’s temperature, pressure, and density are also key physical parameters. As well, tidal and radiogenic heating, heat transfer, the dynamics of aqueous and solid phases (ices and rocks), and radiation are physical processes affecting habitability in Europa’s ocean. Nothing about the known or estimated properties of Europa’s ocean (temperature, pressure, salinity, pH) preclude the possibility of Earth-like microscopic life (Hand et al. 2009).

**Fig. 11:**
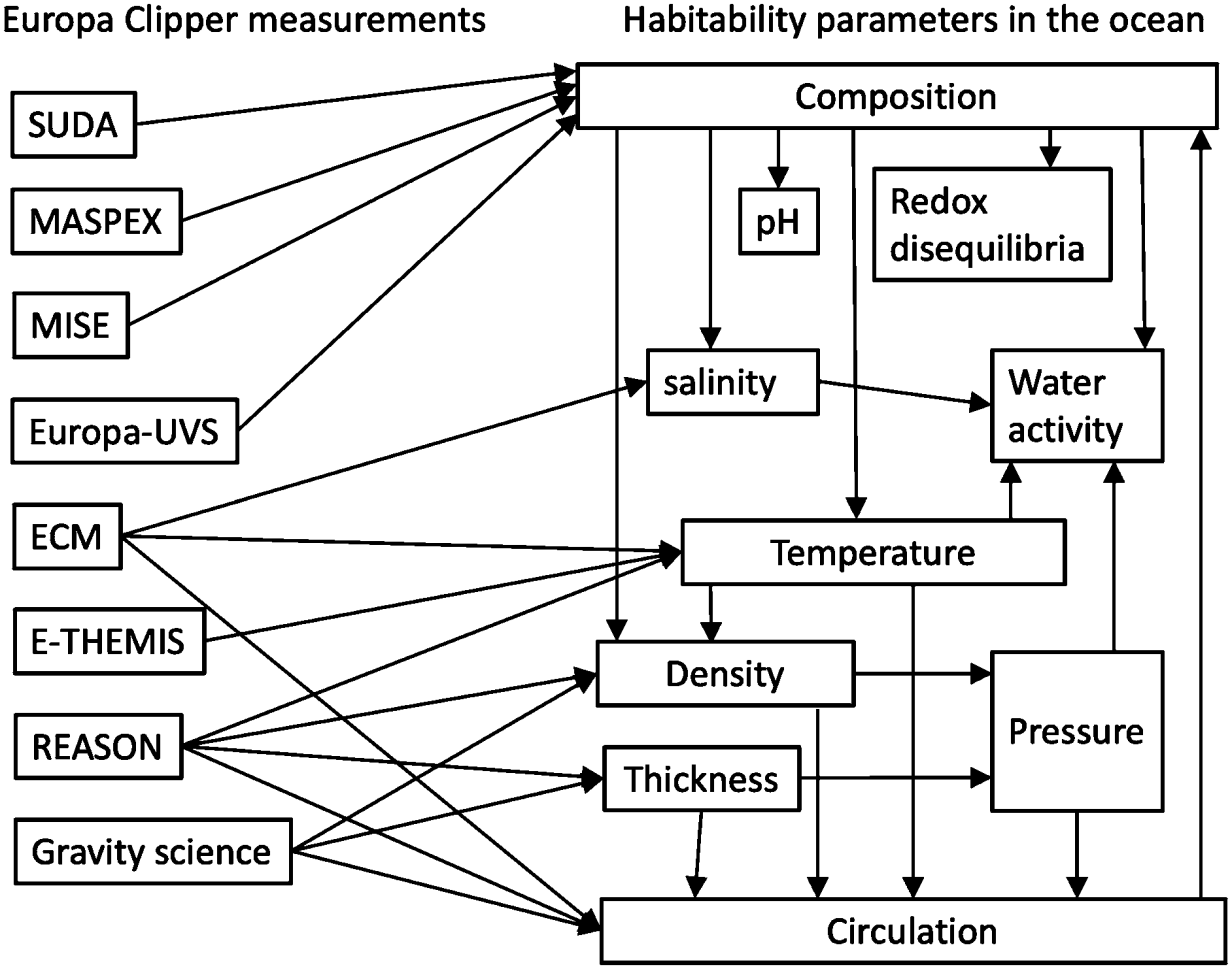
A subset of habitability parameters in the ocean that may be inferred from the synthesis of Europa Clipper measurements. The arrows show the directions of data flow to constrain the parameters. For example, ECM, E-THEMIS, and REASON will contribute measurements that will constrain the temperature of the ocean by measuring the temperature-dependent electrical conductivity of the ocean, and by constraining the composition and thickness of the ice, and thus the temperature at the ice-ocean interface

Although the Europa Clipper investigations will not probe the ocean directly, these parameters will be constrained by the planned in situ and remote measurements (illustrated in very general terms in Fig. 11). In situ measurements will be made of gasses, ions, grains, and electromagnetic properties in atmospheric and possible plume materials by ECM, PIMS, MASPEX, and SUDA; and by remote measurements of optical, RF, gravitational, and thermal properties of surface and subsurface materials by E-THEMIS, EIS, REASON, G/RS, MISE, and Europa-UVS. Table 1, descriptions in Sect. 1, and the following discussion provide details on the measurements by the investigations to obtain further information on the composition and activity at Europa that can shed light on the composition of the ocean.

The habitability of Europa’s ocean will depend on the degree to which, and frequency with which, the ocean water cycles through the mantle and crust at the ocean floor and within the ice in the icy shell (Hand et al. 2009). As the present paper has emphasized repeatedly, fluid cycling through the ocean floor potentially yields solutions rich in reductants — provided the interior geology of Europa continues to supply freshly exposed mantle rocks, or volcanic products derived from partial melting of such mantle rocks, which can react with ocean water. And cycling within the icy shell almost certainly introduces abundant oxidants, which are produced radiolytically at the surface (as seen today), into the ocean. Both oxidants and reductants are needed to power life (e.g., Fig. 9). Hand et al. further state “Based on the need for energy, liquid water, and a suite of biologically essential elements, the prime habitats for life on Europa (were life to exist) are likely to be at the seafloor–ocean interface and at the ice–ocean interface. Here chemical energy and useful compounds and elements may combine with liquid water to provide the conditions needed for life.” That said, this does not mean that the bulk of the ocean in between is necessarily a biological (or habitability) desert, but a well-mixed ocean will not possess the type of gradients that characterize the “prime habitat” interfaces above, either on Europa or on Earth (i.e., seafloor and surface).

The degree to which Europa Clipper can determine the oxidation state of the bulk ocean will be critical. That is, will the ocean turn out to be highly reduced, dominated by the flux of reductants produced by hydrothermal activity (and alteration of mafic and ultramafic minerals), or highly oxidized, overwhelmed by the flux of H_2_O_2_, O_2_, and sulfate from the surface? Neither of these scenarios are ideal for habitability, although in the reduced case, methanogenesis is still possible (utilizing volcanic CO_2_; McCollom 1999). At least theoretically, both oxidants and reductants can be produced radiolytically at Europa’s surface (Chyba and Phillips 2001) and in pore fluids in the rocky crust (Bouquet et al. 2017). But something more intermediate would not only be more habitable, but possibly could implicate mediation by biology. The speciation of nitrogen in the ocean, whether highly reduced (ammonia and ammonium), highly oxidized (nitrate and nitrite), or more intermediate (nitrate and organic compounds), would be telling (Hand et al. 2009). No confirmed detection of nitrogen on Europa’s surface has been made as of this writing (e.g., Carlson et al. 2009), but the suite of compositional measurements by Europa Clipper, of both geologically recent surface materials, and of possible plume materials, holds great promise in this regard, and others, as amplified below.

As an example of combining measurements to constrain the ocean composition, the Europa Clipper will map the distribution of oxidizing compounds on the surface through remote sensing by MISE in the infrared wavelength range and Europa-UVS in the ultraviolet wavelength range (of the surface and atmospheric particles) alongside in situ measurements of sputtered and potential plume material by ECM, PIMS, SUDA, and MASPEX. When taken in context with constraints on the extent of overturning or other renewal of the ice obtained through observations by EIS, REASON, and E-THEMIS investigations, the measurements will inform on the chemical makeup of the subsurface ocean. Over Europa’s history, if large fluxes of oxidizing materials from the ice migrated into the ocean, the ocean’s pH would potentially be at acidic levels only amenable to extremophiles (Pasek and Greenberg 2012). If the ocean acidity reached pH < 6, hydrothermal activity might have sequestered sulfate into seafloor minerals (Tan et al. 2021), so Europa Clipper measurements indicating an acidic ocean relatively free of sulfates might argue for past hydrothermal activity. Conversely, hydrothermal activity may buffer the ocean pH toward more neutral or even alkaline values, as on the Earth and Enceladus (Zolotov and Shock 2001; cf. Fifer et al. 2022). Figure 11 outlines how key habitability parameters in the ocean are expected to be constrained from Europa Clipper measurements.

The abundances of major ions (e.g., Cl^−^, $\mathrm{SO}_{4}^{2-}$, Na^+^, Mg^2+^), neutral solutes (e.g., CH_4_, CO_2_, H_2_, Ar, SiO_2_), and organic compounds in oceanic water—the ocean’s composition—could be inferred from the composition of surface and sputtered materials (MISE, Europa-UVS, EIS, SUDA, MASPEX)—accounting for fractionation processes occurring within the ice shell and during the sputtering process—and from any plume gasses and grains if putative ocean-sourced plumes are sampled by SUDA, MASPEX and Europa-UVS. Surface materials include fresh plume deposits, if present, other endogenic material emplaced by geological processes, e.g., ridges, (spreading) bands or chaos, and exogenic particles such as micrometeorites and sulfur particles from Io. The amounts of surface salts indicated by reflectance spectroscopy could imply a very salty ocean. However, it seems more likely that surface processes have concentrated the salt, and interpretations of Galileo magnetic induction measurements suggest the ocean salinity is not saturated (Hand and Chyba 2007). Europa Clipper measurements will further constrain the origin and processing (e.g., by irradiation or geologic activity) of detected salts and their types and abundances.

The fractionation of Europa’s endogenic materials by geological processing in the icy lithosphere must be carefully assessed, which is one reason why the investigation of Europa’s global geology is a key part of the Europa Clipper mission (Daubar et al. 2024 this collection). Determining which deposits are relatively recent and thus less altered by the harsh radiation environment at the surface will require both detailed morphology and stratigraphic analysis of the surface (EIS, REASON, and MISE), thermal signatures of recent activity (E-THEMIS), and an overall understanding of the thickness of the ice and the degree to which it is convecting (REASON, ECM, PIMS). Fractionation processes include freezing and boiling (in vacuo), radiolytic alteration, exogenic (mainly ionian) pollution of the surface, and the radiolytic and chemical alteration of atmospheric species (Becker et al. 2024 this collection). Surface radiolytic products (e.g., H_2_SO_4_, O_2_, H–_2_O_2_, perchlorates, irradiated salts) detected remotely (MISE, Europa-UVS, EIS color) and in situ in sputtered grains (SUDA) and gasses (MASPEX) together with geological mapping and tectonic reconstructions (EIS, REASON) would provide clues to the fluxes of those species to the ocean. Inferences from any plume samples of the compositions of major ions, and data on CO_2_/H_2_O ratios, would inform about the pH, salinity, and water activity. The importance of salinity for habitability is discussed in detail in Sect. 1.

The abundances of major ions (MISE, SUDA) constrain the ocean’s temperature, density, and viscosity, all of which affect fluid dynamics and mass/heat diffusion in the ocean and at the ocean–rock interface. Thermal mapping of the surface (E-THEMIS) and constraints on the icy shell thickness and brine occurrence within the shell (REASON, MISE, EIS, ECM) also contribute to constraints on the oceanic temperature by providing clues to the ocean’s salinity and to the temperature at the ice-ocean interface. Magnetometer data that identify the amplitude and phase of electromagnetic induction from saline ocean waters (ECM, PIMS) will constrain our knowledge of the thickness and conductivity of the ocean layer (Kivelson et al. 2000). Knowledge of the ocean’s electrical conductivity can, in turn, provide constraints on the ocean’s salinity (Hand and Chyba 2007; Vance et al. 2021). Global oceanic circulation might also create magnetic induction signals, but whether such signals can be detectable by the ECM investigation is subject to further modeling (Vance et al. 2021). Similarly, lateral circulation in the ocean might also be constrained from the topography of the ice–ocean interface (REASON, G/RS) (Zhu et al. 2017; Lobo et al. 2021) and/or surface topography data (EIS). Any interpretations of the ice–ocean interface would need to be grounded in an understanding of tidal heating within the ice (REASON, G/RS, E-THEMIS) (Ojakangas and Stevenson 1989).

The combination of measurements and inferences of ice and ocean thicknesses from ECM, REASON, and G/RS observations, together with composition-based data on density, will constrain the pressure profile within the ocean. Using this information, the density profile of the ocean can be further refined based on knowledge of the ocean’s composition—the salinity of the ocean determines the temperature at the ice–ocean interface and the compressibility of the ocean as a function of depth. The convective adiabat of the ocean depends on both of these properties (e.g., Vance et al. 2021), and might thus be inferred from Europa Clipper’s combined objectives of understanding Europa’s composition, geology, and interior. Further, interpretation of ocean circulation would be grounded in understanding the salinity and pressure conditions of Europan seawater, and whether the anomalous thermal expansion of water affects fluid motion and thermal transport near the ice-ocean interface (Melosh et al. 2004; Kang et al. 2022).

### At the Seafloor

In imagining the habitability of Europa’s seafloor, the analogy of microbial communities around deep-sea hydrothermal systems on Earth is tempting (e.g., McCollom 1999). Pore fluids could potentially support sub-seafloor microbial ecosystems within the rock, as have been found on Earth (Huber et al. 2006; Edwards et al. 2005). However, the extent and nature of water rock interaction depends on the changing balance of volcanic and chemical alteration of the seafloor. The high pressures at the base of the ocean might squeeze shut pathways in the rock relative to the permeabilities observed at mid-ocean-ridge depths (∼2.5 km) in the Earth’s ocean (Klimczak et al. 2019). Conversely, cooling of Europa’s rock under a shallower gravitational gradient could create porosity to 10 s of km depth (Vance et al. 2007, 2016), and this effect could vary with latitude if tidal heating in the rock is significant (Běhounková et al. 2021). On Earth, fracture-assisted hydrothermal circulation at mid-ocean ridges extends to depths of at least 10 km (or 300 MPa) (e.g., Solomon et al. 2003; de Martin et al. 2007), so hydrothermal circulation is plausible to depths below Europa’s seafloor (the seafloor pressure likely doesn’t exceed 200 MPa) in excess of 25 km (cf. Sect. 2.5).

Interactions between the ocean and seafloor depend on the geodynamical state of the silicate interior and are thus also key for understanding Europa’s habitability (Dombard and Sessa 2019; Běhounková et al. 2021). Fluid circulation through the seafloor and upper mantle, either as high-temperature hydrothermal activity driven by volcanism (Bland and Elder 2022; Lowell and DuBose 2005) or through low-temperature, exothermic hydration of olivine and pyroxene,i.e., serpentinization, could provide energy to sustain metabolism through the production of hydrogen, methane, and other simple organics (e.g., Vance et al. 2007, 2016).

**Fig. 12:**
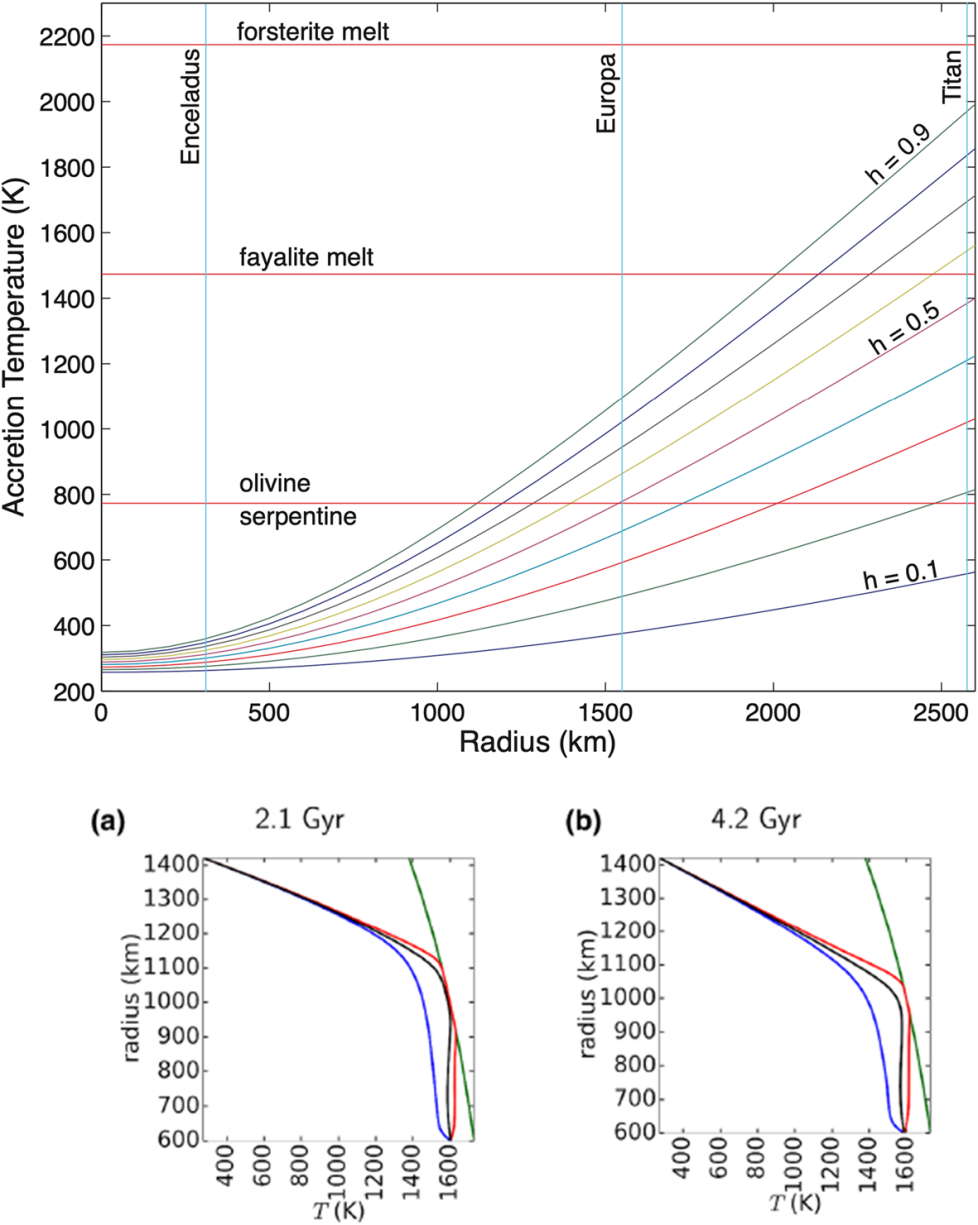
**Top**: Bulk accretion temperatures vs object radius, after McKinnon and Zolensky (2003). For a retention factor of $h=0.4$ (which depends on accretion rate and planetesimal size), Europa’s temperature exceeds the dehydration temperature of serpentine minerals (800 K). **Bottom**: Predicted mantle temperatures in Europa at 2.1 and 4.2 Gyr after accretion, including tides. From Běhounková et al. (2021). Adopted with permission from John Wiley and Sons; blue: minimum, red: maximum, black: average, green: solidus temperature)

We note that a “cold” or limited-energy evolution of Europa would mean limited fluxes into the ocean in recent epochs of hydrogen-rich materials from either low- or high-temperature hydrothermal activity. If interior temperatures never exceeded the dehydration temperature of serpentine-group minerals (800–1000 K at Europa mantle pressures; e.g., Bose and Navrotsky 1998), then low temperature hydration of rock by serpentinization may have run to completion (Vance et al. 2016). However, the current understanding of Europa’s rocky interior (Becker et al. 2024 this collection; Roberts et al. 2023 this collection; Greeley et al. 2004; Schubert et al. 2009) strongly suggests that it was heated enough in its history, through radiogenic and accretional heating alone (Fig. 12). The sum of heat sources plausibly would have been sufficient to release bound water and thus for renewed serpentinization (retrograde metamorphism) to continue to be an important process as a complement to volcanic activity, possibly to the present day.

The extent and duration of any volcanic activity is also still an open question. Contemporary volcanism from tidal heating could be limited, for example if radiogenic material partitioned into a silicate crust in an earlier episode of heating (Moore and Hussmann 2009), which could limit the ability of the crust to subsequently heat and tidally deform. But a warm silicate interior cannot be excluded based on current knowledge, and could be producing several TW of heat. Běhounková et al. (2021) show that mantle tidal dissipation could be a substantial source of internal heating during periods of enhanced eccentricity. If the crust and upper mantle was heated sufficiently, then chemical alteration of the upper 10 s of km of crust and mantle will affect the ion and volatile speciation of hydrothermal fluids relative to the input fluids from the overlying ocean. This chemical imprint may be present in materials analyzed by the Europa Clipper mission. On Earth, the effect of this alteration depends on the residence time of the fluids in oceanic crust, the temperature, pressure, and the corresponding rates of the water-rock reactions (Fisher and Wheat 2011). These qualities in turn depend on the composition and permeability of the rocks (e.g., Vance et al. 2007; Glein and Waite 2020; Melwani Daswani et al. 2021). The extent of alteration is likely also affected by the composition of the ocean and pore fluids. The pH and salinity of the liquid will affect speciation and dissolution of mantle rock (Zolotov and Shock 2004) as well as the rates of water-rock interaction (Lamadrid et al. 2017).

To constrain the seafloor activity and understand its contribution to habitability, the Europa Clipper science team will need to combine the measurements from all its investigations. In addition to the investigations of the internal thermophysical structure and composition by G/RS, REASON, ECM, and PIMS, sampling of Europa’s surface materials could provide clues to the composition of suboceanic rocks and sediments as well as water-solid reactions at the ocean floor. Sampling ^40^Ar and ^4^He (MASPEX) would constrain the rock types, intensity of water-rock interactions, and the extent of radioactive decay of K, U, and Th that affects radiolysis of water and its possible impact on the redox state of oceanic water (e.g., Bouquet et al. 2017). Detecting sulfates, organic compounds, and H_2_ could suggest available energy sources for sulfate-reducing organisms, while detecting H_2_ together with inorganic C species (carbonates, CO_2_) could indicate an energy source for methanogens (McCollom 1999; Zolotov and Shock 2004; Barge and White 2017) or methanotrophs (White et al. 2020). Some of these volatile species (e.g., CO_2_, CH_4_, NH_3_, H_2_) of seafloor/oceanic origin could be trapped in the ice shell and some be incorporated, and even brought to the surface, as clathrates (Hand et al. 2007; Bouquet et al. 2019). Europa Clipper can assess the inventory of ice bound clathrates through combined composition investigations of surface and any plume materials (MISE, Europa-UVS, SUDA, MASPEX), coupled with geological investigations (EIS, MISE, E-THEMIS, REASON) to assess any influence of clathrates on the mechanical properties of the ice.

## Europa Clipper as a Precursor for a Future Landed Mission

While the search for life will likely require access to Europa’s subsurface, a lander or similar in situ mission will be the next logical step in the exploration of this ocean world (Phillips et al. 2021). A landed mission could provide ground-truth for Europa Clipper’s remote sensing observations, as well as detailed measurements to address key questions relating to the physical and chemical processes that have shaped Europa through time, its present-day state, the ability to host current or past habitable environments, and the potential for the emergence of life on Europa. Irrespective of whether the next mission to Europa follows the Europa Lander mission concept framework of Hand et al. (2022), a landed mission to Europa will benefit enormously from the data collected by Europa Clipper (Hand et al. 2020). In particular, the collection of reconnaissance data will be important for identifying potential landing sites that satisfy criteria for both science value (e.g., show evidence of recent activity and materials sourced from the subsurface for interrogations of biosignatures and habitability) and where it is safe to land with extant technologies (Sect. 5 of Daubar et al. 2024 this collection; Phillips et al. 2021).

The careful design of the Europa Clipper spacecraft and science operations enables observations of the surface concurrently and/or for overlapping spatial regions and associated atmospheric and space environments. Therefore, measurements by the remote sensing investigations at high resolutions can be correlated with those by the in situ investigations, providing a full picture of the potential for scientific discovery, as well as mapping for terrain relative navigation and the presence or lack of hazards in different regions for a landed mission. Of particular interest for the terrain relative navigation are the stereo visual images that will be obtained by EIS WAC (≤22 m pixel scale) for solar incidence angles between 30 and 60 degrees over at least 40, 2 km × 4 km-sized regions. EIS NAC data will provide information on surface hazards larger than 2–3 m diameter. A potential extended Clipper mission would also provide opportunities for targeted, follow-on observations to further advance science value and landing safety assessments of potential landing sites, and re-observe discoveries from the prime mission that could add potential landing sites. Furthermore, because a landed mission would provide ground-truth for Europa Clipper’s gamut of remote sensing observations, these could then be used more confidently for reconnaissance on future orbiter missions.

Europa Clipper will be the first NASA mission to follow the ocean world exploration pathway outlined in the NASA Roadmap to Ocean Worlds (Hendrix et al. 2019), while also addressing the many ocean-worlds objectives in the 2023–2032 Planetary Science and Astrobiology Decadal Survey (National Academies 2022). The roadmap notes that Europa Clipper will close the remaining gaps related to understanding the energy balance of the moon and whether it possessed the physio-chemical conditions for life, paving the way for in situ exploration by a landed mission (Phillips et al. 2021). The NASA Astrobiology Program’s Research Coordination Network for Ocean Worlds similarly proposes that a surface landed mission is the next compelling step in exploring this ocean world, emphasizing the key habitability and contextual science that such a mission might return (Howell and Pappalardo 2020). Additionally, Europa Clipper and potential follow-on missions feed forward into future studies of the comparative planetology of other ocean worlds such as Titan, Enceladus, and possibly Triton, to help us understand what factors lead to the diversity of planetary bodies we see today. Following Europa Clipper’s overarching goal, what we learn at Europa will aid in constraining which other confirmed and potential ocean worlds are most likely to host current habitable (if not inhabited) environments.

## Conclusion

NASA’s Europa Clipper mission is the first mission designed to investigate the habitability of an icy world, building on lessons from previous missions exploring the outer Solar System. Europa’s ocean, ice shell, geology, composition, space environment, recent activity, and astrobiological potential are all linked. Assessing whether Europa harbors the conditions conducive for life is a huge scientific endeavor involving the synthesis of a multitude of measurements and cooperation among a team of experts spanning many scientific disciplines. The Europa Clipper mission will assess Europa’s habitability through measurements by the suite of investigations to achieve three primary science objectives involving its ice shell, composition, and geology, and will also investigate the cross-cutting science related to current activity, radiation, geodesy, and sites of high science interest for a future lander. The specific science objectives of the mission are to: (1) characterize Europa’s ice shell, ocean, and any subsurface water, including heterogeneity, ocean properties, and the nature of surface–ice–ocean exchange; (2) understand Europa’s composition and chemistry; and (3) understand the formation of surface features and characterize localities of high science-interest. The mission will also search for evidence of recent or current activity, notably plumes and thermal anomalies, and characterize these if present. Europa Clipper will also perform geodetic and radiation measurements, and will assess high-resolution, co-located observations at sites. Habitability assessments will overarch these objectives, drawing from all the investigations, as outlined in Table 1. With simultaneous collection of data coupled with models of Europa’s ocean, chemistry, evolution, and space environment Europa Clipper will perform an integrated assessment of Europa’s habitability. The mission will also gain a further understanding on the tidal energy available and where it is distributed in Europa, if Europa’s environment supplies chemical compounds suitable for sustaining life, and what drivers exist (chemical or physical factors, energetic particles, etc.) for providing chemical disequilibria. The habitability of Europa over time will also be considered through knowledge gained from the Europa Clipper mission. The entirety of Europa, its ice-shell, ocean, seafloor, and their interfaces, will be surveyed with the suite of complementary instrument investigations on the Europa Clipper mission to assess their potential as habitable environments.

Jupiter orbit insertion for Europa Clipper is anticipated in 2030. Work continues toward achieving the best integrated Europa scientific models to assess the habitability of this remarkable moon, and to begin the next exciting phase of ocean world exploration.
